# FEZ1 Forms Complexes with CRMP1 and DCC to Regulate Axon and Dendrite Development

**DOI:** 10.1523/ENEURO.0193-20.2021

**Published:** 2021-04-15

**Authors:** Jie Yin Chua, Shi Jun Ng, Oleksandr Yagensky, Erich E. Wanker, John Jia En Chua

**Affiliations:** 1 Department of Physiology, Yong Loo Lin School of Medicine, National University of Singapore, Singapore 117593, Singapore; 2 Department of Neurobiology, Max Planck Institute for Biophysical Chemistry, Göttingen 37077, Germany; 3 Max Delbrück Center for Molecular Medicine, Berlin-Buch 13092, Germany; 4 LSI Neurobiology Programme, National University of Singapore, Singapore 117456, Singapore; 5 Healthy Longevity Translational Research Program, Yong Loo Lin School of Medicine, National University of Singapore, Singapore 117456, Singapore; 6 Institute of Molecular and Cell Biology, Agency for Science, Technology and Research (A*STAR), Singapore 138673, Singapore

**Keywords:** axon, CRMP1, DCC, dendrite, development, FEZ1

## Abstract

Elaboration of neuronal processes is an early step in neuronal development. Guidance cues must work closely with intracellular trafficking pathways to direct expanding axons and dendrites to their target neurons during the formation of neuronal networks. However, how such coordination is achieved remains incompletely understood. Here, we characterize an interaction between fasciculation and elongation protein zeta 1 (FEZ1), an adapter involved in synaptic protein transport, and collapsin response mediator protein (CRMP)1, a protein that functions in growth cone guidance, at neuronal growth cones. We show that similar to CRMP1 loss-of-function mutants, FEZ1 deficiency in rat hippocampal neurons causes growth cone collapse and impairs axonal development. Strikingly, FEZ1-deficient neurons also exhibited a reduction in dendritic complexity stronger than that observed in CRMP1-deficient neurons, suggesting that the former could partake in additional developmental signaling pathways. Supporting this, FEZ1 colocalizes with VAMP2 in developing hippocampal neurons and forms a separate complex with deleted in colorectal cancer (DCC) and Syntaxin-1 (Stx1), components of the Netrin-1 signaling pathway that are also involved in regulating axon and dendrite development. Significantly, developing axons and dendrites of FEZ1-deficient neurons fail to respond to Netrin-1 or Netrin-1 and Sema3A treatment, respectively. Taken together, these findings highlight the importance of FEZ1 as a common effector to integrate guidance signaling pathways with intracellular trafficking to mediate axo-dendrite development during neuronal network formation.

## Significance Statement

Guidance cue-dependent elaboration of axons and dendrites toward their target neurons is a critical step in the formation of neuronal circuits during brain development. The elongating neurites require a constant supply of biomolecules, but it remains unclear how guidance cues cooperate with intracellular transport. Here, we show that the kinesin-1 adapter fasciculation and elongation protein zeta 1 (FEZ1) forms complexes with collapsin response mediator protein (CRMP)1 or deleted in colorectal cancer (DCC), which are downstream effectors of the Sema3A and Netrin-1 signaling pathway, respectively. FEZ1-deficient neurons not only exhibit abnormal axons and dendrites, they were also unresponsive to Sema3A-dependent or Netrin-1-dependent regulation of axo-dendritic development. Our results highlight FEZ1 as a key convergence point where guidance cues and intracellular transport integrate to coordinate neuronal process development during neuronal network formation.

## Introduction

Guidance cue-dependent navigation of neuronal processes to their cellular targets contributes to the formation of neuronal networks during brain development (Kim and Chiba, 2004; Kolodkin and Tessier-Lavigne, 2011). Extension and branching of these processes as they migrate toward their final destinations are critically dependent on the delivery and insertion of new biomolecules at the elongation sites (Pfenninger, 2009). While this requires coordination between guidance cue signaling and intracellular transport pathways, how this synchronization is achieved remains poorly understood.

Guidance cues are broadly classified into attractive and repulsive cues. Chemoattractive signaling effected, for instance, via Netrin-1 through its receptors deleted in colorectal cancer (DCC) and Down’s syndrome cell adhesion molecule (DSCAM), is responsible for the attraction of commissural axons toward the midline in the spinal cord (Kennedy et al., 1994; Serafini et al., 1994; Keino-Masu et al., 1996; Ly et al., 2008). Members of the semaphorin family of proteins also mediate attractive signaling. For instance, Sema3C, acting in concert with the neuropilin 1 (Nrp1) receptor, guides axon crossings through the corpus callosum (Niquille et al., 2009). Other members of the semaphorin family participate in repulsive signaling. Exposure of neurons to Sema3A causes neuronal growth cones to shrink and collapse (Luo et al., 1993; Messersmith et al., 1995; Fournier et al., 2000; Dent et al., 2004). Binding of Sema3A to its receptor Nrp1 recruits the collapsin response mediator protein (CRMP) family proteins, including CRMP1, to the Plexin (Plxn) co-receptor and trigger their activation via phosphorylation (Rohm et al., 2000; Deo et al., 2004).

In addition to their roles in navigation, guidance cues also determine neurite branching (Bilimoria and Bonni, 2013; Lanoue and Cooper, 2019). Cortical neurons in *sema3A*^−/−^ and *crmp1*^−/−^ mice exhibited decreased dendritic branching; conversely, hippocampal neurons exposed to Sema3A exhibited increased dendritic growth (Shelly et al., 2011; Makihara et al., 2016). Cortical neurons treated with Netrin-1 or Sema3A also show increased or decreased axonal branching, respectively (Dent et al., 2004), and interfering Netrin-1 signaling by shRNA knock down of either DCC or DSCAM or by the addition of an anti-DCC antibody blocked Netrin-1-dependent axonal branching (Huang et al., 2015; Matsumoto and Nagashima, 2017).

Guidance cue signaling is also thought to coordinate the spatiotemporal insertion and removal of membranes and proteins required for growth cone motility in attractive and repulsive responses (Wojnacki and Galli, 2016). Here, members of the soluble *N*-ethylmaleimide–sensitive factor attached protein receptor (SNARE) protein family play critical roles (Barrecheguren et al., 2017; Ulloa et al., 2018). A complex of VAMP7/Syntaxin-1 (Stx1)/DCC functions in exocytosis in response to Netrin-1 chemoattractive signaling (Cotrufo et al., 2011). Netrin-1-induced aggregation of DCC receptors on the growth cone membrane also triggers VAMP2-mediated exocytosis of vesicles (Bouchard et al., 2008; Gopal et al., 2017; Urbina et al., 2018). Conversely, association of VAMP2 to Nrp1 and PlxnA1 enables endocytosis in response to Sema3A-induced repulsive signaling (Zylbersztejn et al., 2012).

A sustained supply of biomolecules required for the extension of navigating neurites is largely supported by the Kinesin superfamily of microtubule-dependent motor proteins and their corresponding motor adapters (Hirokawa and Takemura, 2005). In the case of Kinesin-1, adapters including fasciculation and elongation protein zeta 1 (FEZ1), syntabulin, and JIP family of proteins function to mediate cargo binding (Gindhart et al., 2003; Su et al., 2004; Sun et al., 2011; Watt et al., 2015). Of these, FEZ1 and syntabulin transport synaptic proteins, including Stx1 and Synaptotagmin-1 (Cai et al., 2007; Toda et al., 2008; Butkevich et al., 2016). Interestingly, while enrichment of syntabulin at neurite tips has not been reported, FEZ1 is highly enriched in the growth cones of developing neurons, suggesting that the Kinesin-1/FEZ1 complex is responsible for delivering biomolecules for incorporation into elongating neurites (Chua et al., 2012). Indeed, using PC12 cells as model, initiation of neuritogenesis was found to strictly depend on FEZ1 expression (Kuroda et al., 1999). Moreover, FEZ1 transport vesicles are highly enriched for proteins with essential roles in neuritogenesis, axon guidance and axon growth (Butkevich et al., 2016). Collectively, these observations indicate that FEZ1-mediated transport is required to support neurite elongation. Furthermore, the presence of guidance cue signaling pathway components (such as CRMP1, VAMP2, and VAMP7) in FEZ1 transport vesicles is intriguing and suggests that these pathways could modulate FEZ1 transport as neurites navigate toward their targets. However, this possibility has not been explored.

Here, we identify a new interaction between FEZ1 and CRMP1 and demonstrate that both proteins colocalize in axons and neuronal growth cones. Strikingly, FEZ1-deficient neurons develop diminished grown cones and axon arborization similar to those seen when CRMP1 function was disrupted. Moreover, developing neurons also exhibited defects in dendrite development in the absence of either protein. Remarkably, FEZ1-deficient neurons showed greater impairment, suggesting the involvement of additional signaling cues in regulating its function. Indeed, FEZ1 puncta colocalizes with VAMP2, the common effector SNARE protein involved in both Sema3A and Netrin signaling. Moreover, FEZ1 is able to form a separate complex with DCC and Stx1 that is required for Netrin-1 signaling. Confirming these observations, we show that FEZ1-deficient neurons become unresponsive to Netrin-1-dependent or Sema3A-dependent regulation of axo-dendritic development. Collectively, these observations highlight a critical role of FEZ1 in both axon and dendrite development and maturation and as a convergence point to effect the downstream signaling of two established guidance cues.

## Materials and Methods

### cDNA and constructs

Plasmids expressing full-length, truncated and various phosphomutant FEZ1, and Myc-Stx1 were obtained as described previously (Chua et al., 2012). Plasmids expressing full-length CRMP1 were obtained by shuttling the entry vector containing human CRMP1 cDNA (obtained from Prof Erich Wanker; Max Delbrück Center for Molecular Medicine) into the destination plasmids pFLAG-CMV2 (Max Delbrück Center for Molecular Medicine) and pcDNA6.2/N-EmGFP-DEST (Invitrogen). Truncated cDNAs for CRMP1 were generated by PCR and inserted into pENTR/D-TOPO entry vectors. Primer sequences used are as follows (5′ to 3′): amino acids 1–462 forward, CACCATGTCGTACCAGGGCA; amino acids 1–462 reverse, TCAGTTGACGTTGATGTTTCCGTC; amino acids 445–572 forward, CACCATGGTCATCAGCCAGGGCAAG, amino acids 445–572 reverse, TCAACCGAGGCTGGTGATGT; amino acids 465–572 forward, CACCATGGGCCGCTTCATTCCG; amino acids 465–572 reverse, TCAACCGAGGCT GGTGATGT. The entry vector containing human DCC cDNA was generated by PCR using pCMV-DCC as a template (a gift from Bert Vogelstein, Addgene plasmid #16459; http://n2t.net/addgene:16459; RRID: Addgene_16459). The entry vector containing the Plxn A1 Extracellular Domain was generated by PCR using PlxnA1-Fc-His (a gift from Woj Wojtowicz, Addgene plasmid #72122; http://n2t.net/addgene:72122; RRID: Addgene_72122). Primer sequences used are as follows (5′ to 3′): forward, CAC CAT GCC ACT GCC ACC TCT G, reverse, TCA CAG TGT CAG CAG GCT GTC CGA AT. The corresponding mammalian expression plasmids were then obtained by shuttling the entry plasmids with the destination plasmids. mCherry-Nrp1 and VAMP2-GFP were gifts from Guido Serini (Addgene plasmid #21934; http://n2t.net/addgene:21934; RRID:Addgene_21934) and Reinhard Jahn, respectively.

### Cell culture and transfection

Human embryonic kidney 293 (HEK293) cells were cultured in DMEM supplemented with 10% fetal bovine serum (FBS) and 0.5% penicillin/streptomycin under normal growth conditions (37°C, 5% CO_2_) and are routinely tested for mycoplasma contamination. Transfection was conducted 1 d after plating, when HEK293 cells were at 70–90% confluency. Polyethylenimine (PEI; Sigma-Aldrich) was used as the transfection reagent in a 1:3 DNA:PEI ratio.

### Co-immunoprecipitation

Twenty-four hours after transfection, cells were lysed with cold HEPES lysis buffer (50 mm HEPES, 150 mm NaCl, 1 mm EDTA, and 1% Triton X-100, pH 7.2) supplemented with the cOmplete EDTA-free Protease Inhibitor Cocktail (Sigma-Aldrich) at 4°C for 15 min. Cell lysates were then clarified by centrifugation at 10,000 × *g* for 10 min at 4°C. The pull-down was performed using GFP-Trap agarose beads (Chromotek) or Pierce Protein A/G agarose beads (Thermofisher Scientific). Clarified lysates were incubated with 10 μl of washed GFP-Trap bead slurry for 1–3 h at 4°C with rotation, or with 2 μl of α-FLAG for 3–4 h, followed by 30 μl of washed Protein A/G agarose bead slurry for 1–2 h at 4°C with rotation. After three washes with HEPES lysis buffer, proteins were eluted using 2× NuPAGE LDS sample buffer (Thermofisher Scientific) supplemented with DTT, followed by heating at 70°C for 10 min.

### Immunoblotting

Protein samples were resolved by SDS-PAGE. Semi-dry transfer using Trans-blot Turbo (Bio-Rad) was used to transfer proteins onto a nitrocellulose membrane. Membranes were blocked for 30 min using 5% skimmed milk in TBST (150 mm Tris-HCl, 1.5 M NaCl, and 0.5% Tween 20, pH 7.4) at room temperature, followed by primary antibody incubation overnight at 4°C. After three washes, membranes were incubated with their respective secondary antibody at a 1:4000 dilution for 1 h at room temperature. Antibody dilutions and their sources are summarized in Table 1. After another three washes, membranes were developed using SuperSignal West Dura Extended Duration Substrate (Thermofisher Scientific) and imaged using the Azure Biosystem C300 (Azure Biosystems). Quantification of protein bands was performed using ImageJ (FIJI). Signal intensities of FEZ1 or CRMP1 were normalized against those of the loading control actin. The normalized band intensities of each protein for each experimental group were then divided by the corresponding intensities from the uninfected (control) group and expressed as a percentage compared with the uninfected group. For co-immunoprecipitation, signal intensities of the coimmunoprecipitated protein were normalized to the signal intensity of the immunoprecipitated binding partner to obtain a semi-quantitative measure of binding strength.

**Table 1:** Antibodies used for immunoblotting and immunofluorescence in this study

	Dilution		Source	RRID
	WB	IF		
Primary antibody			
Mouse α-FLAG M2	1:1000	-	Sigma-Aldrich	AB_262044
Mouse α-CRMP1	1:1000	1:200	Santa Cruz Biotechnology	AB_10846086
Mouse α-VAMP2	-	1:200	Synaptic Systems	AB_887811
Rabbit α-βactin	1:1000	-	Synaptic Systems	AB_11042458
Rabbit α-FEZ1	1:1000	1:400	In house	
Rabbit α-GFP	1:20,000	-	Synaptic Systems	AB_887725
Mouse α-MAP2	-	1:1000	Merck	AB_477256
Rabbit α-MAP2	-	1:1000	Synaptic Systems	AB_2147096
Mouse α-Myc	1:1000	-	In house	
Guinea Pig α-Tau	-	1:400	Synaptic Systems	AB_1547385
Secondary antibody			
Goat α-mouse IgG (H + L)-HRP conjugate	1:4000	-	Bio-Rad	AB_11125547
Goat α-rabbit IgG (H + L)-HRP conjugate	1:4000	-		AB_11125142
Donkey α-mouse IgG (Cy2)	-	1:200	Jackson ImmunoResearch	AB_2340827
Donkey α-mouse IgG (Cy3)	-	1:200		AB_2315777
Donkey α-rabbit IgG (Cy2)	-	1:400		AB_2340612
Donkey α-rabbit IgG (Cy3)	-	1:200		AB_2307443
Donkey α-guinea pig IgG (Cy5)	-	1:400		AB_2340462

### Primary neuronal culture

Adult female Wistar rats with their corresponding litters were purchased from the Centre for Animal Resources and housed in temperature controlled (23 ± 1°C), individually ventilated cages on a 12/12 h light/dark cycle (7 A.M. to 7 P.M.) with access to food and water. Rats were acclimatized for 4 d before the start of experiments. All procedures were in accordance with the Principles of Laboratory Animal Care and approved by the Institutional Animal Care and Use Committee.

Primary hippocampal neurons were prepared from freshly isolated hippocampal tissue from newborn rat brains and immersed in dissection solution (1.5 mm CaCl_2_, 4.9 mm KCl, 0.2 mm NaH_2_PO_4_, 11 mm MgCl_2_, 0.3 mm MgSO_4_, 130 mm NaCl, 2.7 mm NaHCO_3_, 0.8 mm Na_2_HPO_4_, 22 mm HEPES, and 5 mm glucose, pH 7.32). Dissociation of neurons was performed by incubation with 1 ml trypsin for 30 min at 37°C, followed by neutralization with 2 ml 5% serum media (MEM Eagle modified, 21 mm D-glucose, 200 mm L-glutamine, MEM-vitamin, Mito + serum extender, and 5% FBS). To triturate, digested tissue was passed through a fire-polished Pasteur pipette until large tissue fragments were dispersed. Neurons were pelleted by centrifugation at 500 × *g* for 5 min. After resuspending in serum media, cells were plated on PDL-coated coverslips in wells containing DMEM/F12 supplemented with B27.

### Immunofluorescence

Primary hippocampal neurons were fixed using 4% paraformaldehyde in PBS (Santa Cruz Biotechnology). Neurons were first permeabilized by incubation with 0.3% Triton X-100 for 10 min, followed by blocking for 30 min using 10% normal goat serum (NGS) in PBS. Primary and secondary antibody solutions were prepared using 10% NGS as a diluent as summarized in Table 1, which were then incubated with the coverslips in a dark humidified chamber for 1 h at room temperature in succession. After each incubation, coverslips were washed before proceeding to the next step. After incubation with Hoescht (Invitrogen) diluted 1:10,000 in water for 10 min, coverslips were then mounted onto a glass slide using Fluoro-Gel (Electron Microscopy Sciences) and stored. Images of stained neurons were acquired using the 63× oil-immersion objective on the Axio Observer (Zeiss).

Line scan analyses, Pearson correlation coefficient (PCC) analyses, Sholl analyses, neurite tracing and image segmentation were performed using ImageJ (FIJI), and growth cone area was calculated using Zen. To obtain PCC values, the EzColocalization plugin was used (Stauffer et al., 2018). Merged images containing only the desired growth cones or axons were split into individual channels and input into the plugin. Growth cones were only selected if they could be traced via a continuous axon back to the cell body. Negative control values were obtained by flipping the image in the green channel horizontally. Line scan analyses were performed essentially as described previously (Barszczewski et al., 2008). Briefly, regions of interest (three lines of 10 pixels width and 115 pixels length) drawn randomly across regions that correspond to the proximal, middle and distal regions from the axon, and growth cones to obtain signal intensities. For growth cones, the lines were spread out as evenly as possible to cover the entire growth cone. For axons, the first line was drawn at the end of the axon before it becomes a growth cone, the third line was drawn at the start where it projects from soma and the second line was drawn between the first and third lines (equidistant where possible). Colocalization is deemed to occur when both channels display closely correlating similar peaks and troughs. For axon analyses, neurons were doubly stained for MAP2 and Tau. Only continuous processes that were concurrently Tau^+^ and MAP2^–^ were taken as axons and used for subsequent analyses. Axon branches were defined as protrusions measuring at least 20 μm (Winkle et al., 2014). For analyses of FEZ1 or CRMP1 sgRNA-treated neurons, only neurons negative for staining of either protein (confirmed by immunofluorescence staining) were included in the analyses.

### CRISPR-Cas9 system to knock down CRMP1 and FEZ1 expression

Guide RNAs (gRNAs) targeting human FEZ1 gene (5′-AATCAGCTTCAAGTCCATGG-3′) were designed using the online CRISPR design tool (http://crispr.mit.edu/). gRNAs for CRMP1 (5′-CGACTTCGACGCCTACAGCG-3′) were designed using CRISPOR (Haeussler et al., 2016; Concordet and Haeussler, 2018). CRISPOR scores for both gRNA sequences are summarized in Table 2. The Luc gRNA sequence was used as control (5′-CCGGGCTTTAACGAATATGA-3′). All gRNAs were inserted into LentiCRISPRv2 plasmid (Addgene; plasmid #52961; RRID:Addgene_52961) at the BsmBI restriction enzyme site using the GeCKO protocol (Sanjana et al., 2014; Shalem et al., 2014). Viruses were produced as described previously (Yagensky et al., 2019). Briefly, 13 × 10^6^ HEK293 cells were seeded and grown under normal conditions. A day later, 10 μg of transfer plasmid, 5 μg of pMDLg/pRRE (Addgene; plasmid #12251; RRID:Addgene_12251), 2.5 μg of pRSV-rev (Addgene; plasmid #12253; RRID:Addgene_12253), and 2.5 μg of pMD2.G plasmids (Addgene; plasmid #12259; RRID:Addgene_12259) were transfected into HEK293 cells in 3% FBS DMEM using the PEI transfection reagent in a 1:3 DNA:PEI ratio. After 4–6 h of incubation, media were changed into 20 ml of DMEM/F12 (Thermofisher Scientific) and incubated for an additional 24 h. To harvest the lentiviruses, media from each plate of cells were collected and spun down at 1000 rpm for 5 min at 4°C. The supernatant was passed through a 0.45 μm filter and saved as unconcentrated viruses. For viral concentration, 15 ml of filtered supernatant was concentrated at 3220 × *g* for 30 min at 4°C in an Amicon Ultra 15-ml centrifugal filter (Merck). Primary neurons were infected with lentiviruses 1 d after plating.

**Table 2:** CRISPOR scores for FEZ1 and CRMP1 gRNA sequences

#guideId	FEZ1	CRMP1
targetSeq	AATCAGCTTCAAG TCCATGGAGG	CGACTTCGACGC CTACAGCGTGG
mitSpecScore	73	96
cfdSpecScore	86	98
offtargetCount	154	13
targetGenomeGeneLocus	exon:Fez1	Exon 1
Doench '16-Score	NotEnoughFlankSeq	65
Moreno-Mateos-Score	NotEnoughFlankSeq	24
Out-of-Frame-Score	NotEnoughFlankSeq	61
Lindel-Score	NotEnoughFlankSeq	-
GrafEtAlStatus	GrafOK	-
grafType		-

### shRNA knock down of FEZ1

shRNA targeting FEZ1 (5′-GAGGACCTCGTGAATGAATTT-3′) or Luc (5′-CGTACGCGGAATACTTCGA-3′) were inserted into the FHUG+W vector (a kind gift from Dr. Oliver Schlueter). Primary rat day *in vitro* (DIV)1 neurons were infected with lentiviruses expressing either shRNA and subsequently fixed at DIV3, DIV7, and DIV14 and stained for Tau and MAP2. Neurons were imaged on a Zeiss Axio Observer S1 microscope. The tiling function was used to obtain images for a complete coverslip. Axon and dendrite lengths were determined using NeuronJ (FIJI).

### Netrin-1 and Sema3A treatment

Six days after infection of primary neurons with lentiviruses to knock down CRMP1 or FEZ1, neurons were treated with 250 ng/ml of Netrin-1 or 250 ng/ml of Sema3A (RnD Systems) and incubated for 24 h in normal growth conditions before fixing and immunostaining was performed. For the untreated control, PBS (vehicle control) was added in place of Netrin-1 and Sema3A.

### Statistical analyses

Prism GraphPad (version 8) was used to perform statistical analyses. To calculate statistical significance, one-way ANOVA with Bonferroni correction was applied when comparing three conditions. Kruskal–Wallis analysis with Dunn’s test was performed when comparing three conditions for populations with non-Gaussian distributions. A two-tailed Mann–Whitney test was used when comparing two conditions directly.

## Results

### The coiled-coil region of FEZ1 interacts with CRMP1, a protein participating in axonal outgrowth and growth cone guidance

**Figure 1.**
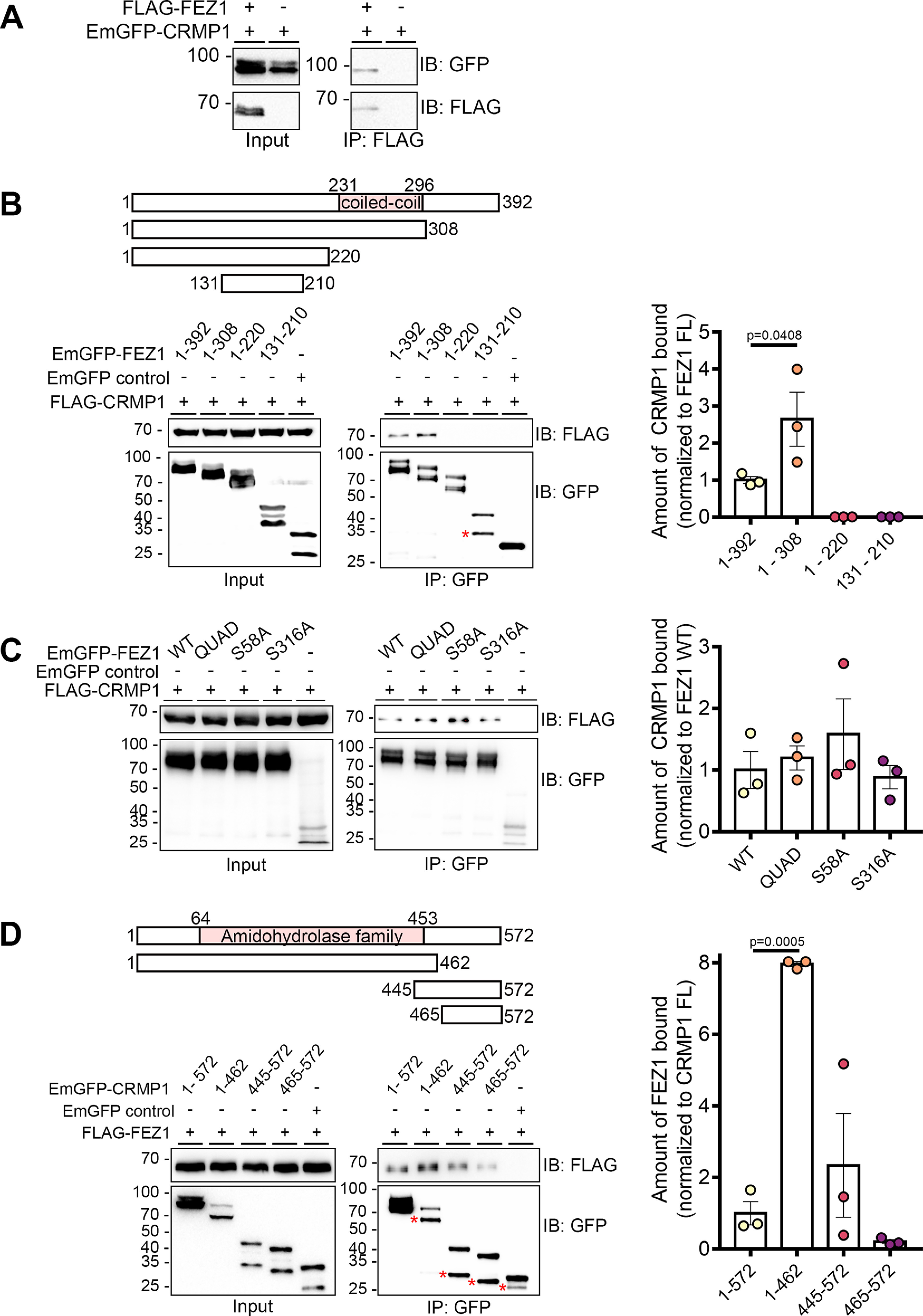
FEZ1 interacts with CRMP1. ***A***, Co-immunoprecipitation of FLAG-FEZ1 FL and EmGFP-CRMP1 FL. Cell lysates of HEK293 transiently expressing FLAG-FEZ1 and EmGFP-CRMP1 were collected and immunoprecipitated using α-FLAG. EmGFP-CRMP1 efficiently co-immunoprecipitated with FLAG-FEZ. ***B***, Mapping of interaction domains on FEZ1. Lysates from HEK293 cells co-expressing the EmGFP-FEZ1 constructs and FLAG-CRMP1 were immunoprecipitated using GFP-TRAP agarose beads. Only FEZ1 constructs containing the coiled-coil domain (amino acids 1–392 and amino acids 1–308) coimmunoprecipitated CRMP1. Truncated FEZ1 lacking its C terminus interacted approximately three times more strongly with FLAG-CRMP1. ***C***, Co-immunoprecipitation of wild-type (WT) or various phosphomutants (QUAD, S58A, or S316A) of FEZ1 tagged to EmGFP, co-expressed with FLAG-CRMP1. Transfected HEK293 cell lysates were collected and immunoprecipitated using GFP-TRAP agarose beads. All FEZ1 phosphomutants co-immunoprecipitated CRMP1. Compared with WT FEZ1, the QUAD and S58A FEZ1 mutants interacted slightly more strongly with CRMP1, but this increase did not reach statistical significance. The S316A FEZ1 mutant displayed a similar interaction level to WT FEZ1. ***D***, Mapping of interaction domains on CRMP1. Lysates from HEK293 cells co-expressing FLAG-FEZ1 and various EmGFP-CRMP1 constructs were immunoprecipitated using GFP-TRAP agarose beads. All versions of CRMP1 were able to interact with FEZ1, suggesting that the amidohydrolase family domain is not critical for this interaction. CRMP1 (amino acids 1–462) bound to FEZ1 more strongly than full-length CRMP1. Red asterisks indicate non-specific bands. Statistical significance was determined using one-way ANOVA. Data were obtained from three independent experiments. All error bars represent SEM. Reciprocal and control coimmunoprecipitations are shown in Extended Data Figure 1-1.

A previous effort to systematically identify new proteins involved in presynaptic function using a yeast 2 hybrid screen uncovered an interaction between FEZ1 and CRMP1 (Chua et al., 2012). CRMP1 has been identified to be present on FEZ1 transport vesicles isolated from the rat brain (Butkevich et al., 2016). To additionally validate that the two proteins interact, we performed co-immunoprecipitation assays using FLAG-FEZ1 and EmGFP-CRMP1 co-expressed in HEK293 cells. Immunoprecipitation of FEZ1 recovered CRMP1 from transfected cell lysates containing both proteins but not in the absence of FEZ1, thereby supporting that FEZ1 forms a complex with CRMP1 (Fig. 1*A*).

10.1523/ENEURO.0193-20.2021.f1-1 Extended Data Figure 1-1 Reciprocal coimmunoprecipitations for FEZ1 and CRMP1 interaction. ***A***, Reciprocal co-immunoprecipitation of EmGFP-CRMP1 truncated constructs. Cell lysates from HEK293 cells transiently expressing FLAG-FEZ1 and various EmGFP-tagged CRMP1 peptides were immunoprecipitated using α-FLAG. Both the upper and lower bands of each EmGFP-CRMP1 construct were co-immunoprecipitated and were absent in their corresponding control coIPs. ***B***, HEK293 cell lysates containing FLAG-Munc18 co-expressing EmGFP-FEZ1 FL or various EmGFP-tagged CRMP1 peptides were immunoprecipitated using GFP-TRAP beads. Only the positive control, EmGFP-FEZ1 FL, was observed to interact with FLAG-Munc18. Download Figure 1-1, DOCX file.

To identify the region of FEZ1 responsible for binding CRMP1, we separately co-expressed full-length FEZ1 or one of the three truncated versions of FEZ1 (amino acids 1–308, amino acids 1–220, and amino acids 131–210) fused to EmGFP with FLAG-CRMP1 in HEK293 cells. Co-expression of EmGFP alone with FLAG-CRMP1 served as a negative control. FLAG-CRMP1 was resolved as a single band of ∼70 kDa on immunoblots. Full-length and truncated versions of FEZ1 were resolved at ∼97, 90, 75, and 45 kDa, respectively, as previously reported (Chua et al., 2012). The protein is phosphorylated at multiple sites, accounting for the appearance of additional minor bands observed (Chua et al., 2012; Butkevich et al., 2016). An unexpected smaller band was observed for the shortest FEZ1 peptide (indicated by an asterisk) that could have arisen as a result of non-specific cleavage. Using GFP-TRAP beads to pulldown the FEZ1 peptides, only FEZ1 fragments containing the coiled-coil domain (amino acids 1–392 and amino acids 1–308) were observed to interact efficiently with FLAG-CRMP1 (Fig. 1*B*). Truncated FEZ1 peptides lacking the coiled coil domain (amino acids 1–220 and amino acids 131–210) did not pulldown CRMP1, indicating that this protein domain is critical for the interaction (Fig. 1*B*). Remarkably, FEZ1 (amino acids 1–308) displayed a greater affinity (∼3-fold) to interact with CRMP1 as compared with full-length FEZ1 (Fig. 1*B*), suggesting that the C-terminal region of FEZ1 might play an inhibitory role in this interaction.

### Phosphorylation of FEZ1 does not regulate its interaction with CRMP1

Four phosphorylation sites (S58, S134, S301, and S316) were previously identified in FEZ1. Abolishing phosphorylation at these sites by mutating them to alanine residues can affect its binding to some of its interaction partners (Chua et al., 2012). To determine whether phosphorylation of these serine residues might also influence the interaction between FEZ1 and CRMP1, we separately co-expressed three EmGFP-FEZ1 phosphomutants [S58A, S316A, and QUAD (S58A, S134A, S301A, S316A)] with FLAG-CRMP1 and used GFP-TRAP beads to pulldown FEZ1 (Fig. 1*C*). All phosphomutants retained their ability to bind CRMP1 (Fig. 1*C*). Thus, the binding between the two proteins is unaffected by FEZ1 phosphorylation, which is similar to what has been reported for its interaction with Stx1 (Chua et al., 2012).

### The CRMP1 amidohydrolase domain is involved in FEZ1 binding

CRMP1 contains a central amidohydrolase family domain comprising of a triosephosphate isomerase-like barrel. The TIM barrel is a common structure involved in many enzyme-ligand interactions (Kurochkina, 2010), suggesting the involvement of this domain in protein interactions. To identify whether this region in CRMP1 is involved in FEZ1 binding, we co-expressed FLAG-FEZ1 separately with three truncated versions of EmGFP-CRMP1 corresponding to its N-terminal and central amidohydrolase family domain (amino acids 1–462) and the C-terminal regions (amino acids 445–572 and amino acids 465–572). CRMP1 was then immunoprecipitated using GFP-TRAP beads. Supporting the previous observations, reciprocal coimmunoprecipitation with EmGFP-CRMP1 full-length also coimmunoprecipitated FLAG-FEZ1 (Fig. 1*D*). Sequential deletion of CRMP1 progressively diminished its interaction with FEZ1, with the exception of CRMP1 (amino acids 1–462) where binding to FEZ1 was significantly increased. Interestingly, the C-terminal CRMP1 alone (amino acids 465–572) also retained residual binding to FEZ1, which was enhanced when a small portion of the conserved amidohydrolase family domain was included (amino acids 445–572). No FEZ1 was detectable in the negative control where CRMP1 was omitted. In addition to the larger bands corresponding to the expected band sizes for each truncated CRMP1 peptide, a smaller accompanying band could also be observed (Fig. 1*D*, asterisks). Reciprocal coimmunoprecipitations with FLAG-FEZ1 coprecipitate both long and short peptides, suggesting that the smaller peptides did not correspond to non-specific interactions (Extended Data Fig. 1-1*A*). Furthermore, as expected, immunoprecipitation of the CRMP1 truncated peptides did not co-immunoprecipitate Munc18 (Extended Data Fig. 1-1*B*) showing that detection of this interaction could not be attributable to non-specific binding. Taken together, these results indicate that the two halves of CRMP1 may possess independent interaction regions for FEZ1 with the amidohydrolase family domain-containing region binding more strongly to FEZ1 (Fig. 1*D*). It is remains unclear why, on their own, each half of CRMP1 appears to exhibit stronger binding as compared with the full-length protein.

### FEZ1 and CRMP1 colocalize in growth cones of developing neurons

The CRMP family of proteins participate in Sema3A signaling and are involved in regulating several aspects of neuronal development (Yamashita and Goshima, 2012). In particular, CRMP1 participates in neurite outgrowth and growth cone dynamics in developing neurons (Higurashi et al., 2012). Likewise, FEZ1 is readily detectable in growth cones of developing hippocampal neurons (Chua et al., 2012). These observations suggest that the FEZ1-CRMP1 complex could function to coordinate delivery of cargoes to neurite tips.

**Figure 2.**
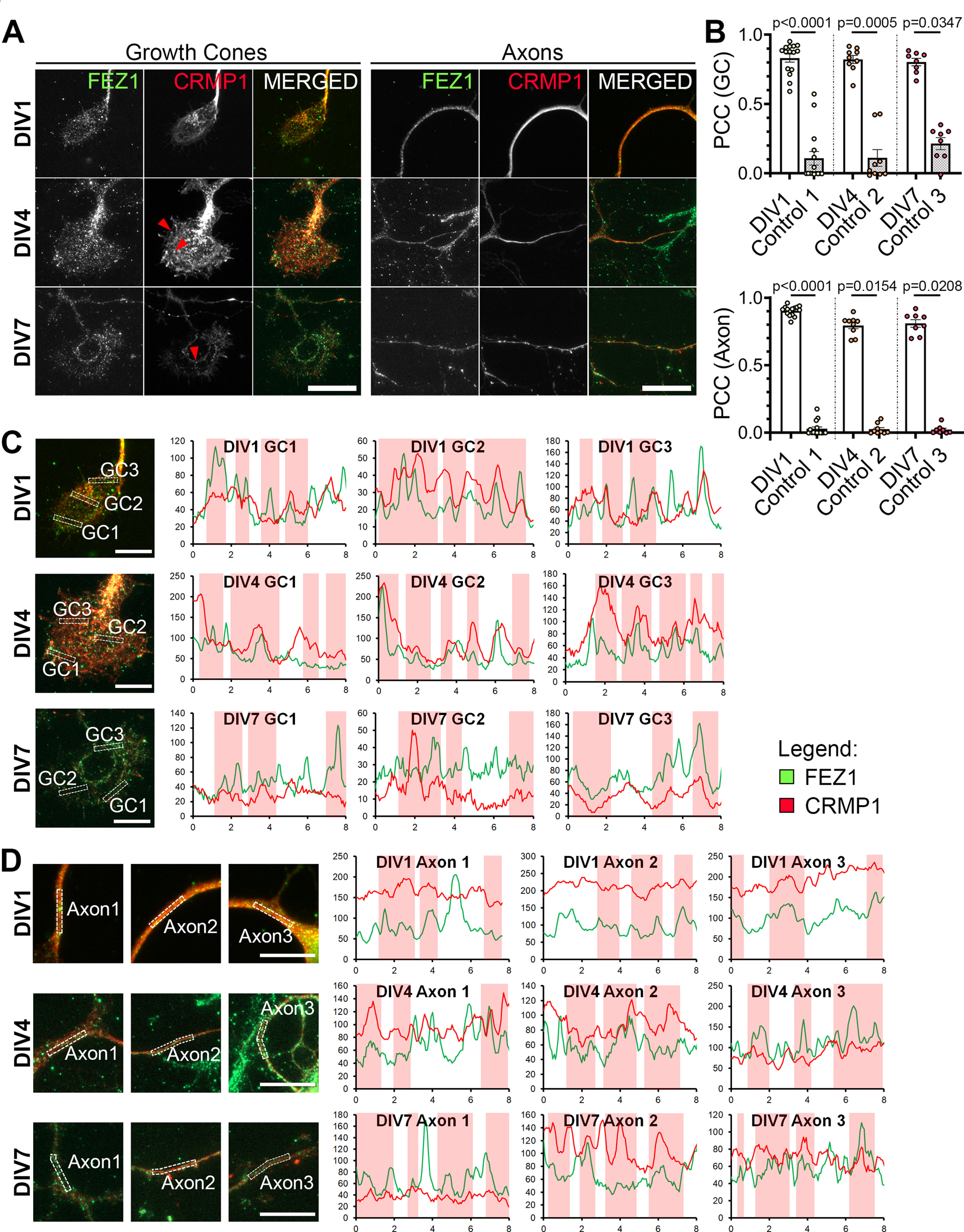
FEZ1 and CRMP1 colocalize in hippocampal neurons. ***A***, Distribution of FEZ1 and CRMP1 in growth cones and axons of hippocampal neurons at 1, 4, and 7 DIV. Puncta for FEZ1 and CRMP1 (red arrowheads) can be observed in the growth cones. Scale bars: 20 μm. ***B***, PCC analysis of colocalization extent of both proteins in growth cones and axons. Control values were obtained by obtaining the PCC with the rotated image of one channel; *n* = 15 (DIV1), *n* = 9 (DIV4), *n* = 8 (DIV7) collected across three independent experiments. Statistical significance was determined using Kruskal–Wallis analysis. All error bars represent SEM. ***C***, ***D***, Line scan analysis for colocalization between FEZ1 and CRMP1 in growth cones and axons respectively. Merged images are shown. *x*- and *y***-**axes, distance (μm) and gray value respectively; axon 1, distal; axon 2, intermediate; axon 3, proximal (relative to cell body). Vertical columns in red represent regions of colocalization. Scale bars: 10 μm.

To determine this, we first examined the localization of both proteins in developing hippocampal neurons. As previously reported, FEZ1 puncta are distributed in cell bodies, axons and growth cones (Fig. 2*A*; Ikuta et al., 2007; Chua et al., 2012). In comparison, CRMP1 shows a more uniform distribution in neurons, especially throughout the length of neurites. As previously reported, CRMP1 is also present in growth cones, with some regions showing punctate distribution (Fig. 2*A*, arrowheads; Higurashi et al., 2012). Colocalization analysis via PCC revealed a significant overlap of both proteins in growth cones and axons. In comparison, the coefficient significantly decreased when rotated images from one channel were used for the analyses (Table 3; Fig. 2*B*). We further examined colocalization of both proteins using line scan analysis. Supporting the previous analysis, correlating signal peaks indicative of colocalization could be observed in growth cones and axons (Fig. 2*C*,*D*, vertical red columns). Together, these results indicate that FEZ1 and CRMP1 can form a complex in axons and, in particular, in growth cones.

**Table 3:** Summary of PCC values (colocalization of FEZ1 and CRMP1)

	Days *in vitro*	PCC	
		FEZ1 + CRMP1	Control
GC	1	0.8311 ± 0.0277	0.108 ± 0.0472
	4	0.8214 ± 0.0275	0.1104 ± 0.0570
	7	0.8024 ± 0.0252	0.214 ± 0.0404
Axons	1	0.9026 ± 0.009	0.02804 ± 0.0134
	4	0.794 ± 0.0222	0.02467 ± 0.0121
	7	0.8094 ± 0.0265	0.0216 ± 0.0107

### Loss of FEZ1 causes growth cone collapse and decreased axonal branching

**Figure 3.**
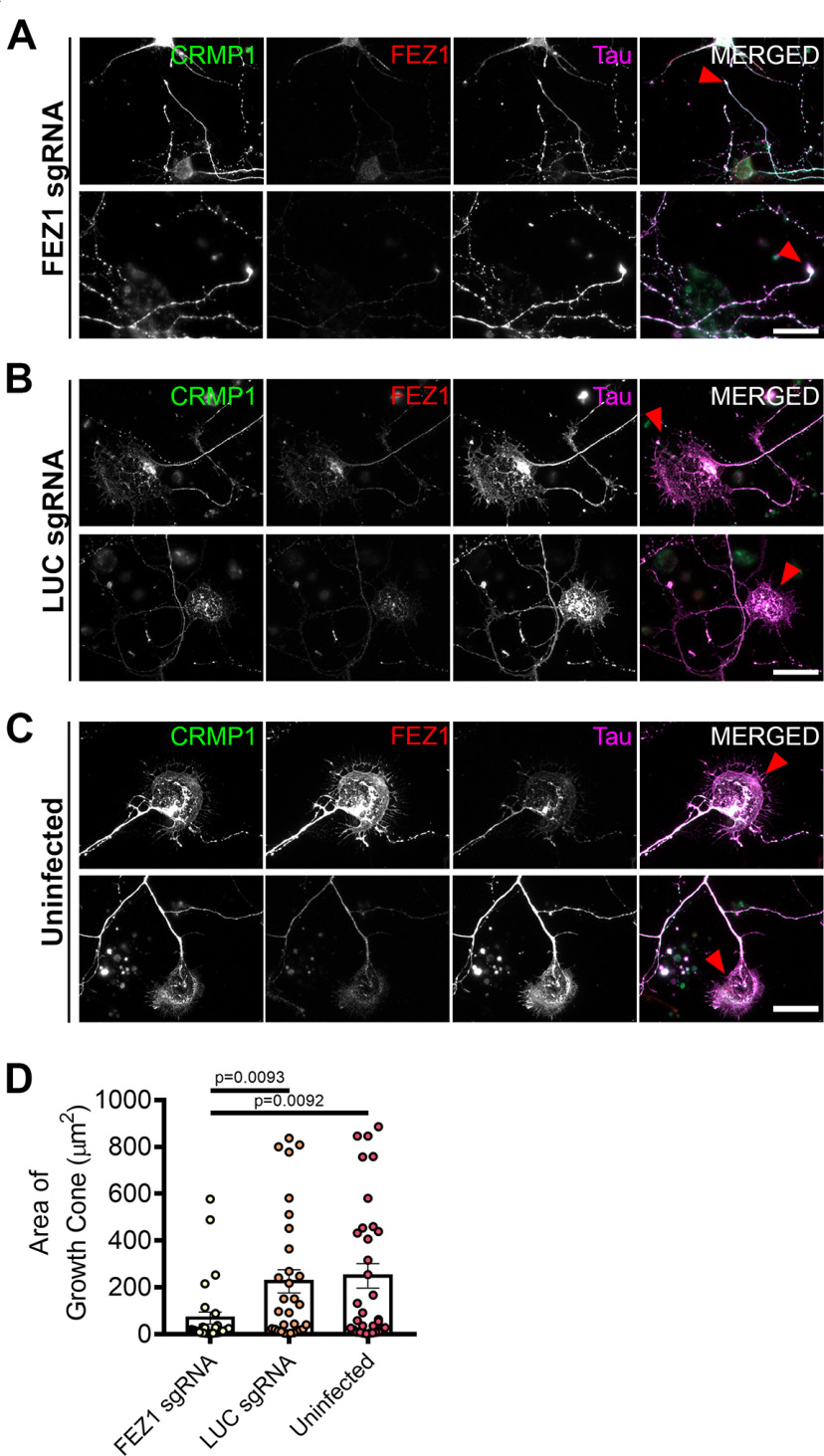
FEZ1-deficient neurons display collapsed growth cones. ***A–C***, Immunostaining against CRMP1, FEZ1, and Tau in hippocampal neurons treated with FEZ1 sgRNA, LUC sgRNA, or uninfected controls, respectively, 6 DPI. Majority of the FEZ1-deficient neurons displayed smaller growth cones compared with the controls (red arrowheads). Scale bars: 20 μm. ***D***, Quantification of growth cone sizes in FEZ1-deficient or control LUC sgRNA and uninfected neurons. On average, growth cones in control neurons were ∼4- to 5-fold larger than those in FEZ1-deficient neurons. Statistical significance was determined using Kruskal–Wallis analysis. FEZ1 sgRNA *n* = 30, LUC sgRNA *n* = 31, uninfected *n* = 33, collected over three independent experiments. All error bars represent SEM. Western blottings for CRIPSR/Cas9-mediated FEZ1 knock-down in neurons are shown in Extended Data Figure 3-1.

To further examine whether the FEZ1/CRMP1 complex might be involved in supporting neurite extension, we employed a lentiviral-based CRISPR-Cas9 system to ablate FEZ1 expression using FEZ1 sgRNAs in developing hippocampal neurons. As a control, we used a sgRNA targeting a scrambled luciferase sequence (LUC sgRNA; Yagensky et al., 2019). Neurons at 1 DIV were infected with the corresponding viruses and lysates immunoblotted to probe for FEZ1 expression. Approximately 80% of endogenous FEZ1 protein was eliminated 6 d post virus infection (DPI; Extended Data Fig. 3-1*A*). Abrogation of FEZ1 expression in individual neurons was first confirmed using immunofluorescence staining (Fig. 3*A*). Only neurons successfully knocked down for FEZ1 were selected for analyses. We could not study the effect of FEZ1 loss in neurons younger than 6 DIV as endogenous FEZ1 could still be detected from 1 to 4 DPI (Extended Data Fig. 3-1*B*).

10.1523/ENEURO.0193-20.2021.f3-1 Extended Data Figure 3-1 Knock down of FEZ1 in rat hippocampal neurons. The Lentiviral CRISPR/Cas9 system was used to knock-down FEZ1 in primary rat hippocampal neurons at DIV1. ***A***, Representative Western blotting showing FEZ1 knock-down in rat hippocampal neurons 6 DPI. FEZ1 levels was reduced by ∼70%. Statistical significance was determined using one-way ANOVA. Data were obtained from three independent experiments. Values displayed represent mean ± SEM. ***B***, Neurons at 1–4 DPI were lysed and probed using an α-FEZ1 antibody. No changes in FEZ1 levels compared to control neurons were observed from 1 to 4 DPI. ***C***, However, at 6 DPI, a clear decrease in FEZ1 levels were observed. Download Figure 3-1, DOCX file.

FEZ1 has previously been found to deliver biomolecules to the growth cones (Chua et al., 2012); and if FEZ1 was involved in CRMP1 transport, a general mislocalization of CRMP1 would have been expected. However, CRMP1 distribution in neurons was largely unchanged in the absence of FEZ1 (Fig. 3*A–C*), indicating that FEZ1 is not critical for its transport. However, using Tau as a label for both axons and growth cones (Mandell and Banker, 1996; Biswas and Kalil, 2018), we observed that growth cones were significantly smaller in FEZ1-deficient neurons but were still present at axon ends in control neurons (Fig. 3*A–C*, red arrowheads). Indeed, the average growth cone area of these neurons was at least 4-fold smaller as compared with the control neurons (Fig. 3*D*).

**Figure 4.**
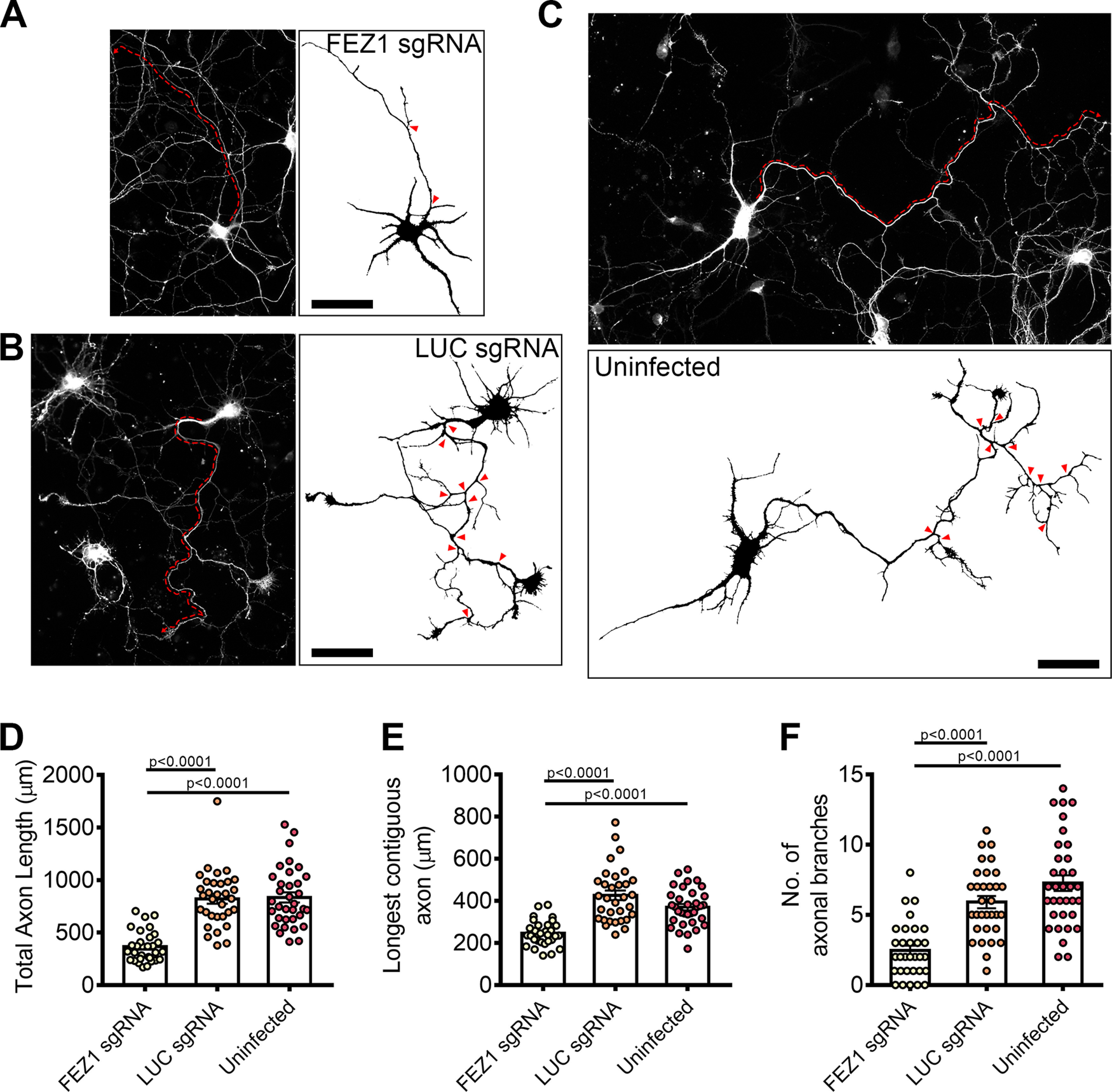
FEZ1-deficient neurons display defects in axonal development. ***A–C***, Tiled images of FEZ1 sgRNA, LUC sgRNA, and uninfected neurons immunostained for Tau. Respective axon traces are shown juxtaposed. Axons of FEZ1-deficient neurons are significantly less branched as compared with control neurons (branch points indicated by red arrowheads). The length of the longest contiguous axon (red dotted lines) in FEZ1-deficient neurons was also shorter than in control neurons. ***D–F***, Quantification of total axon length, longest contiguous axon length, and number of axon branches in FEZ1 sgRNA, LUC sgRNA, and uninfected neurons, respectively. All quantified variables were significantly decreased in FEZ1-deficient neurons as compared with control groups. Statistical significance was determined using Kruskal–Wallis analysis. FEZ1 sgRNA *n* = 31, LUC sgRNA *n* = 32, uninfected *n* = 34, obtained from three independent experiments. Error bars represent SEM. Scale bars: 50 μm. Developmental abnormalities in shRNA-mediated FEZ1 knock-down hippocampal neurons are shown in Extended Data Figure 4-1.

To directly examine whether loss of FEZ1 function directly affected neurite extension, we measured and compared total axon (Tau^+^ and MAP2^–^) length as well as the length of the longest contiguous axon (Fig. 4*A–C*, red dotted lines) between control and FEZ1-deficient neurons. Both parameters were significantly decreased in the latter group (Fig. 4*A–C*, quantifications in *D*,*E*). We also observed decreased axonal branching in FEZ1-deficient neurons as compared with both sets of control neurons (Fig. 4*A–C*, red arrowheads show branch points, quantifications in *F*). Indeed, several neurons lacking FEZ1 displayed no branch points, a phenotype not observed in either set of control neurons surveyed. Using lentiviral shRNA as an alternative strategy to knock down FEZ1 expression, we also observed impairments in axon development where mean axon length was significantly shorter in FEZ1 knocked down neurons (Extended Data Fig. 4-1). These results implicate FEZ1 in axon elongation and arborization during neurite outgrowth.

10.1523/ENEURO.0193-20.2021.f4-1 Extended Data Figure 4-1 shRNA-mediated FEZ1 knock-down in rat hippocampal neurons. ***A***, Representative Western blotting showing FEZ1 knock-down in rat hippocampal neurons. Quantification is shown on the right. ***B***, ***C***, FEZ1 knock-down neurons show axo-dendrite developmental abnormalities. ***D***, Quantification of total axon length and dendrite branches. FEZ1 knock-down neurons show significantly shorter axons and dendrite branching as compared to control neurons. Download Figure 4-1, DOCX file.

**Figure 5.**
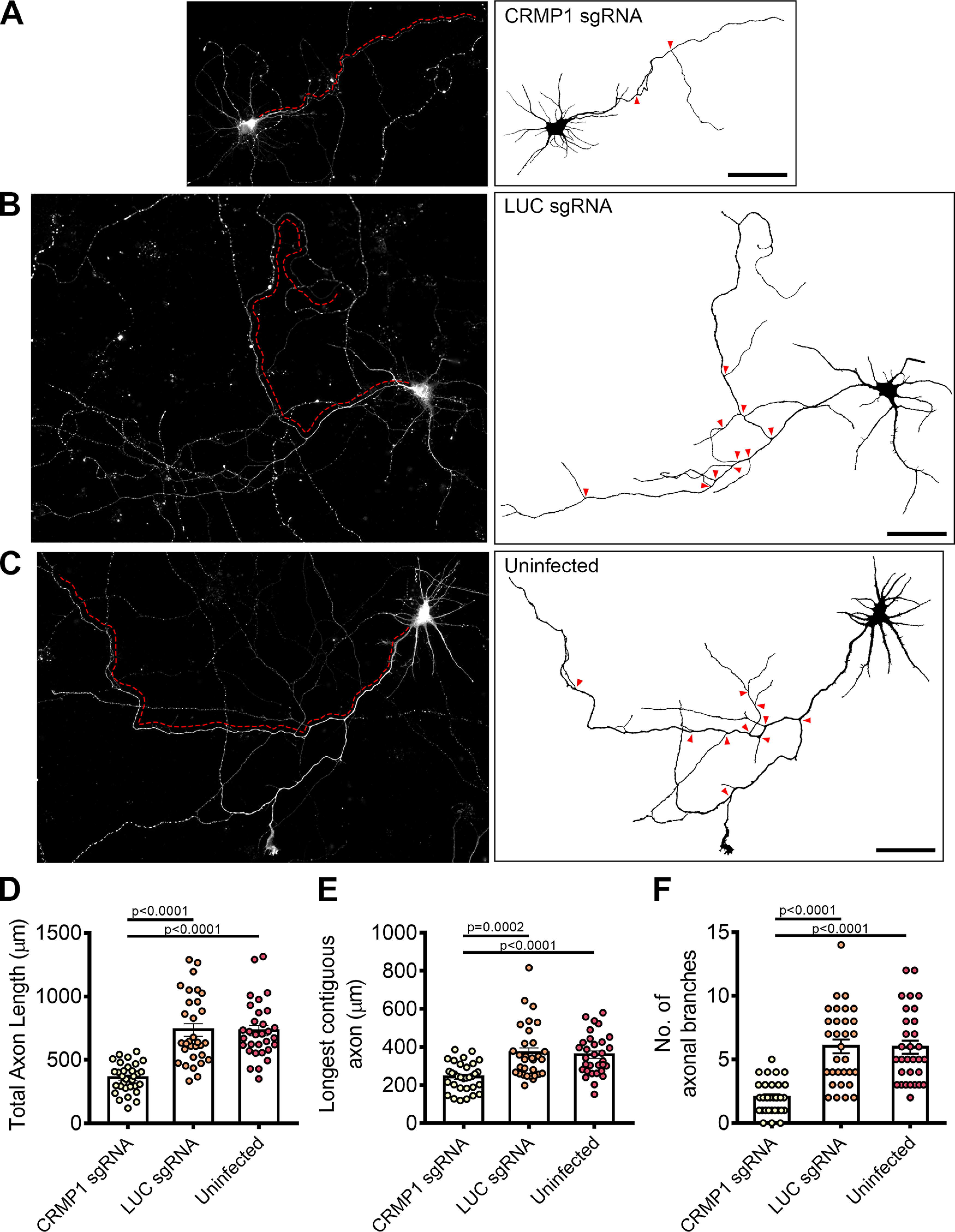
CRMP1-deficient neurons display defects in axonal development. ***A–C***, Tiled images of CRMP1 sgRNA, LUC sgRNA, and uninfected neurons immunostained for Tau. Respective axon traces are shown juxtaposed. Axons of CRMP1-deficient neurons are significantly shorter and less branched as compared with control neurons (branch points indicated by red arrowheads; longest contiguous axon for each neuron is indicated by red dotted line). ***D–F***, Quantification of total axon length, longest contiguous axon length and number of axon branches in CRMP1 sgRNA, LUC sgRNA, and uninfected neurons, respectively. All three variables were significantly decreased in CRMP1-deficient neurons as compared with control groups. Statistical significance was determined using Kruskal–Wallis analysis. CRMP1 sgRNA *n* = 32, LUC sgRNA *n* = 31, uninfected *n* = 31, obtained from three independent experiments. Error bars represent SEM. Scale bars: 50 μm. Western blottings for CRIPSR/Cas9-mediated CRMP1 knock-down in neurons are shown in Extended Data Figure 5-1.

A previous study has shown that CRMP1 is involved in neurite outgrowth of dorsal root ganglion neurons (Higurashi et al., 2012). To investigate whether neurite extension was also affected in CRMP1-deficient hippocampal neurons, we generated a lentiviral vector expressing sgRNA targeting CRMP1 (CRMP1 sgRNA). Following infection, endogenous CRMP1 protein levels were reduced by ∼60% as determined by immunoblotting (Extended Data Fig. 5-1). We repeated the axonal measurements on CRMP1-deficient neurons. As before, CRMP1 knock-down was first confirmed using immunofluorescence, and only these neurons were used for analysis. Indeed, CRMP1-deficient neurons exhibited significantly shorter total axon lengths, shorter contiguous axons and fewer axon branches as compared with control groups (Fig. 5*A–C*, quantifications in *D–F*). Collectively, the phenotypes present in FEZ1-deficient neurons agree well with those reported in neurons when CRMP1 function was perturbed, thereby implicating that FEZ1 and CRMP1 work together in a common pathway.

10.1523/ENEURO.0193-20.2021.f5-1 Extended Data Figure 5-1 Knock down of CRMP1 in rat hippocampal neurons. Representative Western blotting showing knock down of CRMP1 in rat hippocampal neurons 6 DPI. Approximately 60% of CRMP1 was eliminated. Statistical significance was determined using one-way ANOVA. Data were obtained from three independent experiments. Values displayed represent mean ± SEM. Download Figure 5-1, DOCX file.

### FEZ1-deficient neurons display more severe dendritic defects than CRMP1-deficient neurons

In addition to its functions in axon, FEZ1 is also found in dendrites and has been implicated in dendritic development. FEZ1 expression is maintained in adulthood (Sakae et al., 2008) and previous studies in adult hippocampal neurogenesis have shown that shRNA knock down of FEZ1 increased dendritic arborization in dentate granule cells (Kang et al., 2011; Watanabe et al., 2014). To examine whether these effects could be recapitulated during postnatal neuron development, we immunostained wild-type and FEZ1-deficient neurons for MAP2 and performed Sholl analyses as a measure of dendritic development.

**Figure 6.**
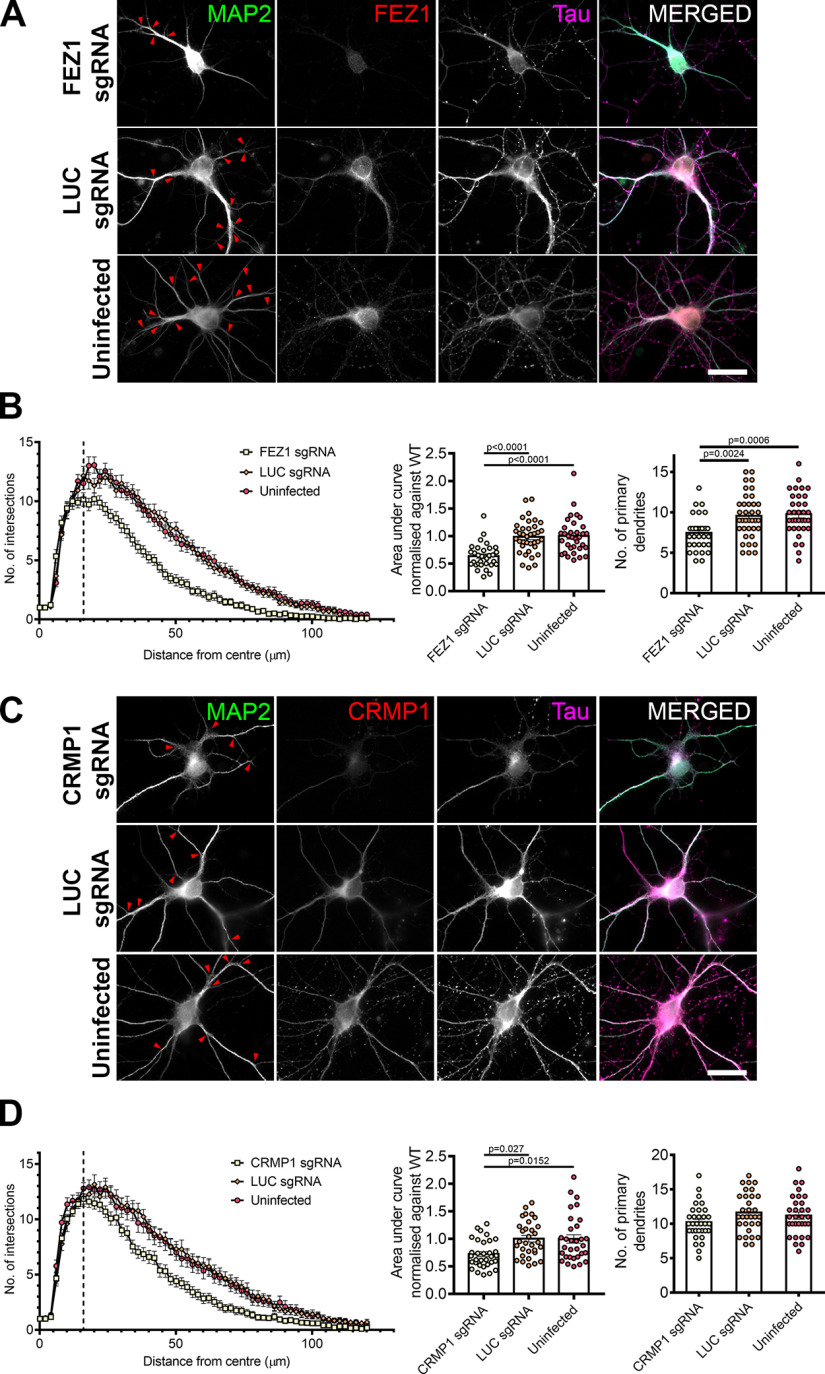
Loss of FEZ1 impairs dendritic development. ***A***, Images of FEZ1 sgRNA, LUC sgRNA, and uninfected neurons stained for MAP2, Tau, and FEZ1 6 DPI. Branch points are denoted by red arrowheads. ***B***, Loss of FEZ1 reduces dendritic branching and number of primary dendrites. Dotted line indicates the peak of intersections for FEZ1-deficient neurons. Statistical significance was determined using Kruskal–Wallis analysis. FEZ1 sgRNA *n* = 32, LUC sgRNA *n* = 37, uninfected *n* = 34, collected over three independent experiments. ***C***, Images CRMP1 sgRNA, LUC sgRNA, and uninfected neurons stained for MAP2, Tau, and CRMP1 at 6 DPI. Branch points are denoted by red arrowheads. ***D***, Loss of CRMP1 reduces overall dendritic branching but does not affect maximum number of crossings and number of primary dendrites. Dotted line indicates the peak of intersections for CRMP1-deficient neurons. Statistical significance was determined using Kruskal–Wallis analysis. CRMP1 sgRNA *n* = 32, LUC sgRNA *n* = 31, uninfected *n* = 31, collected over three independent experiments. All error bars represent SEM. Scale bars: 20 μm.

In stark comparison to what was observed during adult neurogenesis, FEZ1-deficient postnatal developing neurons exhibited fewer MAP2^+^ primary dendrites as compared with wild-type neurons (Kang et al., 2011). Dendritic branching was also reduced (Fig. 6*A*, red arrowheads indicate branch points). Sholl analyses indicated that FEZ1-deficient neurons displayed fewer intersections with concentric circles radiating outwards from the cell body as compared with the controls, confirming that dendritic development in these neurons is significantly impaired (Fig. 6*B*). Likewise, impaired dendritic development was also observed in shRNA-treated FEZ1 knocked down neurons (Extended Data Fig. 3-1). These results indicate that, unlike adult neurogenesis, FEZ1 is required to promote dendritic development and branching in postnatal developing hippocampal neurons.

Dendritic development defects were also present in neurons of Sema3a and CRMP1 knock-out mice (Su et al., 2007; Makihara et al., 2016). In agreement with these reports, we also observed reduced dendritic branching in CRMP1-deficient neurons (Fig. 6*C*, red arrowheads). Sholl analyses confirmed that neurons lacking CRMP1 indeed show less severe defects in dendritic development as compared with FEZ1-deficient neurons (Fig. 6*B*,*D*; Table 4). The mean peak number of intersections for CRMP1-deficient neurons was 11.8 ± 0.47 at distance of 16 μm from the center of the cell body; intersections began to decline thereafter (Fig. 6*D*, dotted line). This peak was comparable in control neurons but the number of intersections in CRMP1-deficient neurons declined faster than in controls (Fig. 6*D*). Contrasting strongly with this, FEZ1-deficient neurons showed a significantly smaller peak (Mann–Whitney two-tailed test, *p* = 0.0369) of 10.2 ± 0.57 at 16 μm (Table 4; Fig. 6*B*, dotted line). Supporting this, the number of primary dendrites was significantly reduced in FEZ1-deficient neurons compared with control neurons, but not in CRMP1-deficicient neurons (Fig. 6*B*,*D*). Moreover, the overall reduction in dendritic network complexity, as represented by the normalized area under curve, was slightly greater in FEZ1-deficient neurons while those in control neurons remained comparable (Table 4; Fig. 6*B*,*D*). Neurons doubly infected with lentiviruses targeting FEZ1 and CRMP1 were not viable, which hindered our attempts to further examine whether there could be an additive effect on dendrite development when both proteins were simultaneously eliminated (data not shown). Nevertheless, the greater reduction in dendritic development arising from loss of FEZ1 as compared with CRMP1 suggested that FEZ1 is likely to participate in other dendritic development signaling pathways in addition to those mediated by Sema3A/CRMP1.

**Table 4:** Summary of Sholl analyses in FEZ1 and CRMP1 knock-down neurons

	sgRNA	Maximum no. of crossings	Distance from centre of cell body (μm)	Total area under curve
CRMP1 knock-down	CRMP1	11.8 ± 0.47	16	0.72 ± 0.044
	LUC	13.16 ± 0.85	20	1 ± 0.0576
	Uninfected	12.9 ± 0.66	18	1 ± 0.075
FEZ1 knock-down	FEZ1	10.15 ± 0.568	16	0.635 ± 0.038
	LUC	12.16 ± 0.8	16	0.98 ± 0.047
	Uninfected	13.05 ± 0.69	20	1 ± 0.055

### FEZ1 colocalizes with VAMP2 in growth cones, a SNARE protein shared by Sema3A and Netrin signaling

In addition to its interaction with CRMP1, FEZ1 interacts with Stx1 in neuronal growth cones, which works downstream of the Netrin-1 receptor DCC to mediate axonal outgrowth in response to Netrin-1 signaling (Cotrufo et al., 2011; Chua et al., 2012). Moreover, VAMP2, a SNARE protein used by both Netrin-1 and Sema3A signaling pathways is present in FEZ1 transport vesicles isolated from rat brains (Zylbersztejn et al., 2012; Butkevich et al., 2016; Gopal et al., 2017; Urbina et al., 2018). Like Sema3A/CRMP1, Netrin-1/DCC signaling is involved in regulating axonal and dendritic development (Kim and Chiba, 2004; Stoeckli, 2018). Thus, we hypothesized that the more severe impairment on dendritic development observed in FEZ1-deficient neurons could be potentially mediated via Netrin-1 signaling pathways.

**Figure 7.**
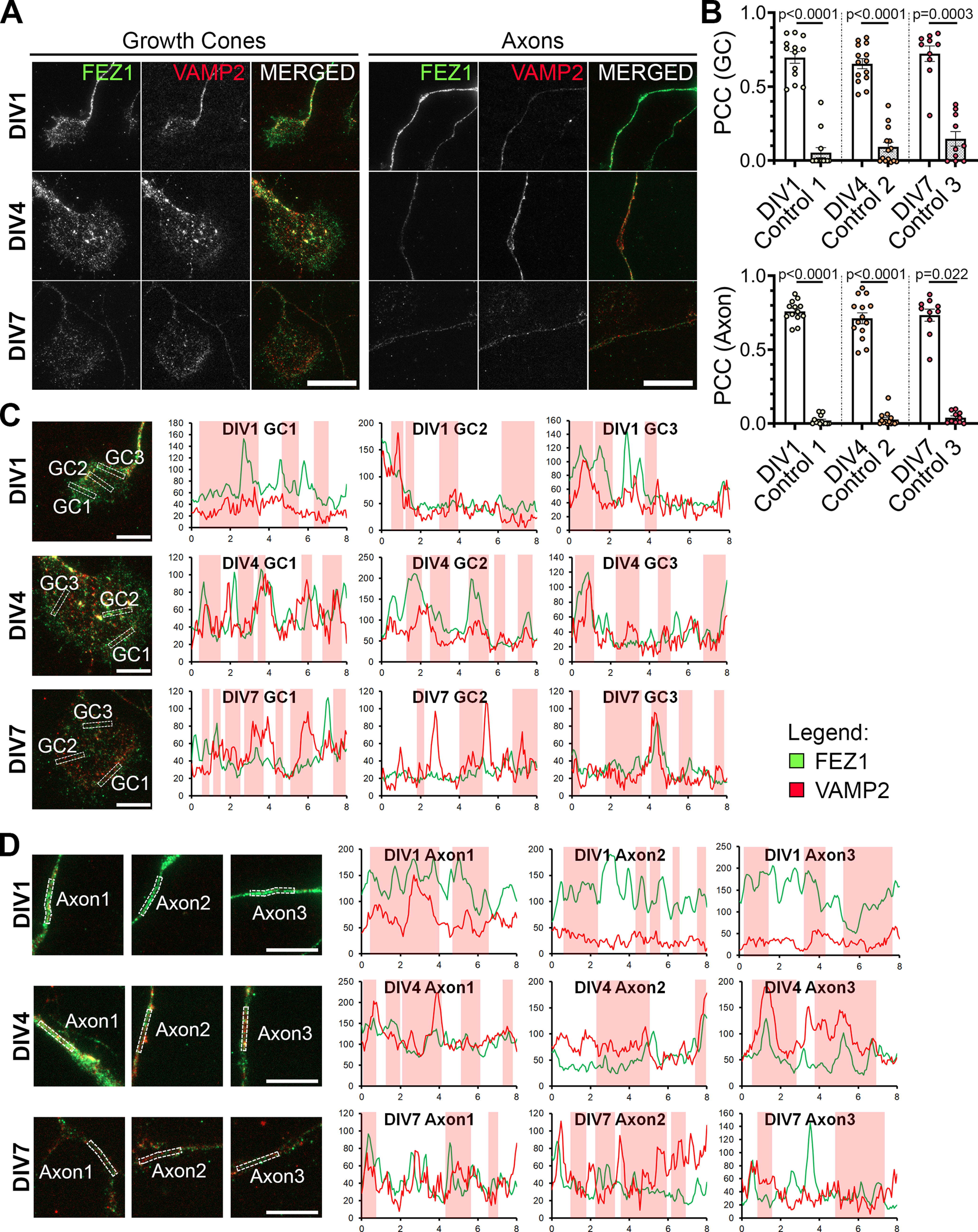
FEZ1 and VAMP2 co-localize in growth cones and axons. ***A***, Immunostaining of FEZ1 and VAMP2 in growth cones and axons of hippocampal neurons at 1, 4, and 7 DIV. Punctate staining of FEZ1 and VAMP2 are observed. Scale bars: 20 μm. ***B***, PCC analysis of fluorescence intensities of both proteins in growth cones. Control values were obtained by measuring the PCC with the rotated image of one channel. DIV1 *n* = 13, DIV4 *n* = 14, DIV7 *n* = 10, collected over three independent experiments. Statistical significance (experimental vs control) was determined using Kruskal–Wallis analysis. All error bars represent SEM. ***C***, ***D***, Line scan analysis for co-localization between FEZ1 and VAMP2 in growth cones and axons. Merged images are shown. *x*- and *y***-**axes, distance (μm) and gray values, respectively; axon 1, distal; axon 2, intermediate; axon 3, proximal (relative to cell body). Vertical columns in red represent regions of colocalization. Scale bars: 10 μm. Additional line scans are shown in Extended Data Figure 7-1.

To determine this, we first investigated whether VAMP2 could also colocalize with FEZ1 in growth cones of developing neurons. In agreement with previous findings, punctate distribution of VAMP2 was detectable along axons and in growth cones (Fig. 7*A*; Baumert et al., 1989; Coco et al., 1999; Cotrufo et al., 2011). The number of VAMP2 puncta increased from DIV1, peaking at DIV4 before decreasing at DIV7. PCC analysis showed partial colocalization of FEZ1 and VAMP2 in growth cones and axons at all stages studied. Correlation significantly decreased when rotated images from one channel were used as controls (Table 5; Fig. 7*B*). Line scan analyses also supported the PCC analyses (Fig. 7*C*,*D*, red vertical columns; additional line scans in Extended Data Fig. 7-1). These results suggest that FEZ1, in concert with VAMP2, could also participate in Netrin-1 signaling. We were unable to examine localization between FEZ1 and VAMP7 because of the lack of a suitable compatible antibody for staining the latter.

**Table 5.** Summary of PCC values (colocalization of FEZ1 and VAMP2)

	Days *in vitro*	PCC	
		FEZ1 + VAMP2	Control
GC	1	0.6972 ± 0.0378	0.05331 ± 0.0324
	4	0.655 ± 0.0328	0.09171 ± 0.0313
	7	0.7232 ± 0.0498	0.1467 ± 0.0466
Axons	1	0.760 ± 0.0192	0.02056 ± 0.00819
	4	0.712 ± 0.0358	0.0272 ± 0.0121
	7	0.7334 ± 0.0386	0.03947 ± 0.0112

10.1523/ENEURO.0193-20.2021.f7-1 Extended Data Figure 7-1 Additional line scan analyses for FEZ1 and VAMP2 in growth cones and axons of developing neurons. ***A***, ***B***, Line scan analyses for growth cones and axons of neurons at 1, 4, and 7 DIV, respectively. Merged images are shown. *x*- and *y***-**axes, distance (μm) and grey values, respectively; axon 1, distal; axon 2, intermediate; axon 3, proximal (relative to cell body). Vertical columns in red represent regions of colocalization. Scale bars: 10 μm (***A***) and 5 μm (***B***). Download Figure 7-1, DOCX file.

### FEZ1 complexes with components of Sema3A and Netrin-1 signaling pathways

**Figure 8.**
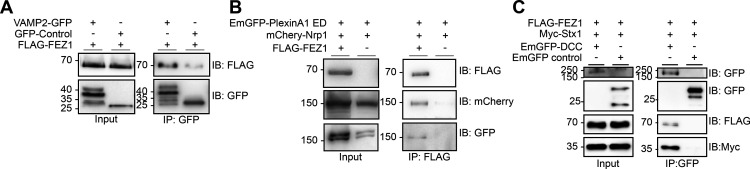
FEZ1 forms complexes with components of Netrin-1 and Sema3A signaling pathways. ***A***, FEZ1 interacts with VAMP2. Lysates from HEK293 cells expressing FLAG-FEZ1 and VAMP2-GFP were immunoprecipitated using GFP-TRAP. FEZ1 co-immunoprecipitates with VAMP2. ***B***, FEZ1 interacts with Nrp1 and PlxnA1. FLAG-FEZ1 was immunoprecipitated using α-FLAG from HEK293 cell lysates expressing FLAG-FEZ1, mCherry-Nrp1, and EmGFP-PlxnA1 ED. Nrp1 and PlxnA1 ED were both co-immunoprecipitated, indicating an interaction between FEZ1 and the Sema3A receptor complex. ***C***, FEZ1 forms a complex with DCC and Stx1. HEK293 cell lysates transiently expressing the three proteins were immunoprecipitated using GFP-TRAP. Both FLAG-FEZ1 and Myc-Stx1 co-immunoprecipitated with EmGFP-DCC, indicating that the three proteins can form a complex. Data were obtained from three independent experiments.

We wondered whether colocalization between FEZ1 and VAMP2 could indicate that the 2 proteins interact. Immunoprecipitation of HEK293 lysates co-expressing VAMP2-GFP and FLAG-FEZ1 using GFP-TRAP show that both proteins can interact (Fig. 8*A*). FLAG-FEZ1 was not detected in control coIPs where VAMP2-GFP was replaced by GFP. We further examined whether FEZ1 could also interact with the Sema3A receptor complex Nrp1/PlxnA1 (Deo et al., 2004; Zylbersztejn et al., 2012). As we were unable to obtain any plasmids expressing full-length PlxnA1, the co-immunoprecipitation experiments were performed using the extracellular domain (ED) of PlxnA1, which is still able to form a complex with Nrp1 (Takahashi et al., 1999). Immunoprecipitation of FLAG-FEZ1 with α-FLAG antibodies co-immunoprecipitated mCherry-Nrp1 and EmGFP-PlxnA1 ED but not when FLAG-FEZ1 was omitted (Fig. 8*B*). This indicated that FEZ1 can also interact with the Sema3A receptor complex.

Binding of Netrin-1 to DCC allows the latter to bind Stx1 to effect subsequent exocytosis for neurite growth (Cotrufo et al., 2011). As FEZ1 colocalizes with Stx1 in growth cones, we wondered whether FEZ1 might also form part of this DCC complex to mediate Netrin-1 signaling (Chua et al., 2012). To examine this, co-immunoprecipitation assays were performed using EmGFP-DCC, FLAG-FEZ1 and Myc-Stx1 in HEK293 cells. Indeed, Stx1 and FEZ1 co-immunoprecipitated with DCC pulled down using GFP-TRAP, but not when DCC was absent, indicating that a complex between FEZ1, Stx1, and DCC can be formed. Together, these data highlight a possible role for FEZ1 in both Sema3A-induced and Netrin-1-induced guidance (Fig. 8*C*).

### FEZ1 is required for both Netrin-1 and Sema3A regulated neurite development

Treatment with Netrin-1 has been reported to increase dendritic arborization during neuronal development (Goldman et al., 2013; Winkle et al., 2016). Similarly, hippocampal neurons exposed to Sema3A also exhibited increased dendritic development (Shelly et al., 2011). To determine whether FEZ1 indeed functions as a common effector downstream of both signaling pathways, we tested how dendritic development of FEZ1-deficient or CRMP1-deficient neurons were affected by Netrin-1 and Sema3A treatments.

**Figure 9.**
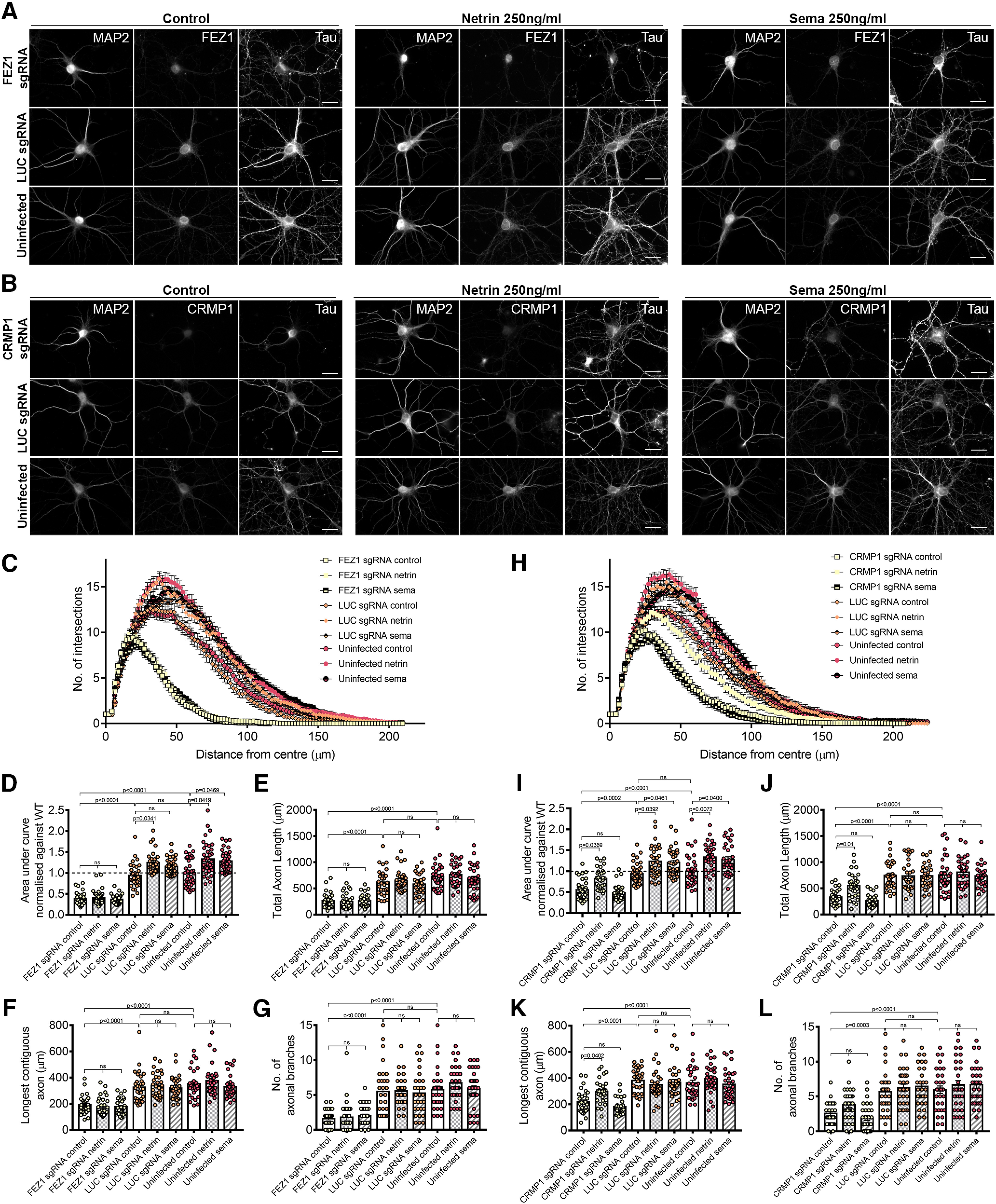
FEZ1 is involved in Netrin-1 and Sema3A-induced dendritic development. ***A***, ***B***, Images of FEZ1 sgRNA or CRMP1 sgRNA, LUC sgRNA, and uninfected neurons treated with Netrin-1 or Sema3A. Neurons were fixed and stained for MAP2, Tau, and FEZ1, or CRMP1 24 h post-treatment. Scale bars: 20 μm. ***C***, ***D***, Sholl analyses and AUC of FEZ1-deficient and control neurons treated with Netrin-1 or Sema3A. Dendritic complexity of control neurons but not FEZ1-deficient neurons were observed to increase after Netrin-1 or Sema3A treatment. FEZ1 sgRNA control *n* = 32, netrin *n* = 35, sema *n* = 33; LUC sgRNA control *n* = 31, netrin *n* = 32, sema *n* = 35; uninfected control *n* = 36, netrin *n* = 38, sema *n* = 36 collected over three independent experiments. ***E–G***, Total axon length, longest contiguous axon, and axonal branching quantifications for FEZ1-deficient and control neurons treated with Netrin-1 and Sema3A. FEZ1-deficient neurons did not respond to either guidance cues. FEZ1 sgRNA control *n* = 31, netrin *n* = 30, sema *n* = 30; LUC sgRNA control *n* = 30, netrin *n* = 30, sema *n* = 30; uninfected control *n* = 30, netrin *n* = 30, sema *n* = 31 collected over more than three independent experiments. ***H***, ***I***, Sholl analyses and AUC of CRMP1-deficient and control neurons treated with Netrin-1 and Sema3A. CRMP1-deficient neurons displayed an increase in dendritic complexity in response to Netrin-1 but not to Sema3A. CRMP1 sgRNA control *n* = 37, netrin *n* = 33, sema *n* = 35; LUC sgRNA control *n* = 37, netrin *n* = 33, sema *n* = 33; uninfected control *n* = 39, netrin *n* = 31, sema *n* = 32 collected over three independent experiments. ***J–L***, Total axon length, longest contiguous axon and axonal branching quantifications for CRMP1-deficient and control neurons treated with Netrin-1 and Sema3A. No significant differences between treated and untreated neurons were observed for all variables measured in the control groups. CRMP1-deficient neurons displayed increased total axonal length and longest contiguous axon length when treated with Netrin-1 but not Sema3A. CRMP1 sgRNA control *n* = 30, netrin *n* = 31, sema *n* = 30; LUC sgRNA control *n* = 31, netrin *n* = 31, sema *n* = 30; uninfected control *n* = 31, netrin *n* = 31, sema *n* = 31 collected over more than three independent experiments. For all analyses, statistical significance was determined using Kruskal–Wallis analysis. All error bars represent SEM.

Neurons infected with FEZ1, CRMP1, and LUC sgRNA were incubated with Netrin-1 or Sema3A for 24 h at 6 DPI. As with previous reports, treatment with either Netrin-1 or Sema3A increased dendritic arborization of neurons in the control groups (Fig. 9*A*,*B*, FEZ1 in *C*,*D*, CRMP1 in *H*,*I*). Since CRMP1 has been shown to mediate Sema3A signaling (Deo et al., 2004; Yamashita et al., 2007), our observation that CRMP1-deficient neurons did not exhibit increased dendritic arborization when treated with Sema3A is in agreement with previous findings. However, when treated with Netrin-1, CRMP1-deficient neurons showed a significant increase in dendritic complexity against untreated controls (Fig. 9*B*,*H*,*I*), indicating that CRMP1-deficient neurons can still respond to Netrin-1 signaling. Strikingly, FEZ1-deficient neurons responded neither to Sema3A nor Netrin-1 (Fig. 9*A*,*C*,*D*). Indeed, the level of dendritic complexity in these neurons was comparable to untreated FEZ1-deficient neurons. Thus, the lack of responsiveness to either stimulus in these neurons indicated that FEZ1, unlike CRMP1, is a common effector of both signaling pathways.

We further investigated whether similar effects could be seen in developing axons. Netrin-1 treatment was previously observed to increase axon length and branching, whereas Sema3A reportedly inhibited axonal development (Shelly et al., 2011; Winkle et al., 2016). We did not observe any changes in total axon length, longest contiguous axon length and axonal branching when control neurons were treated with Netrin-1 or Sema3A likely since the treatments were only applied after axons have developed (Fig. 9*E–G*,*J–L*). While exposure of CRMP1-deficient neurons to Sema3A did not cause any change, treatment of these neurons with Netrin-1 induced a significant increase in total axon length, the longest contiguous axon length and a slight increase in axonal branching (Fig. 9*J–L*). These increases were completely abolished in Netrin-1-treated FEZ1-deficient neurons that were also unresponsive to Sema3A treatment (Fig. 9*E–G*). Taken together, these results indicate that FEZ1 acts at least downstream of Netrin-1 to regulate axon development.

## Discussion

Proper development of neuronal networks in the developing brain requires precise guidance of extending neuronal processes to their targets. This extension must be supported by the delivery of new building materials to support neurite outgrowth. However, how the requisite coordination between these two processes occurs remains unclear. Here, we identified and characterized the involvement of the FEZ1/CRMP1 complex in the development of axons and dendrites. Eliminating FEZ1 expression resulted in neurons with impaired axon and dendrite development. Dendritic impairment in FEZ1-deficient neurons was more severe than those observed in CRMP1-deficient neurons, uncovering the possibility that another signaling pathway could be involved in regulating FEZ1 function. Indeed, we show that FEZ1 colocalizes with VAMP2, a SNARE protein shared by both Sema3A and Netrin-1 signaling pathways and that ternary complexes of FEZ1 could be formed with Stx1 and DCC as well as Nrp1 and Plexin A1. Significantly, developing dendrites of FEZ1-deficient neurons failed to respond to Sema3A and Netrin-1 stimulation. In addition, their axons were also unresponsive to Netrin-1 treatment. Collectively, these results demonstrate the critical involvement of FEZ1 in axon and dendrite development and highlight its role as a common effector to integrate guidance cue pathways to the development of axons and dendrites during the generation of neuronal networks.

Previous studies demonstrated the involvement of FEZ1 in neuritogenesis and its role in transporting proteins involved in synaptic transmission and neurodevelopment (Fujita et al., 2007; Ikuta et al., 2007; Chua et al., 2012; Butkevich et al., 2016). However, it was unclear whether guidance pathways might work via FEZ1 to direct cargoes to sites of neurite elongation. Our finding that FEZ1 can form complexes with components of the Sema3A and Netrin-1 signaling pathways indicate that guidance cues can work via the adapter to achieve this. In support of this, FEZ1-deficient neurons were unable to maintain growth cone morphology and a smaller proportion of these neurons analyzed possessed large growth cones compared with control neurons (Fig. 3). Moreover, in agreement with its role in the axonal transport, FEZ1-deficient neurons also exhibited defects in axon development (Fig. 4; Toda et al., 2008; Chua et al., 2012; Butkevich et al., 2016). These phenotypes are also manifested in CRMP1-deficient neurons, indicating that both proteins are important for growth cone function (Higurashi et al., 2012). We further demonstrated that both FEZ1-deficient and CRMP1-deficient neurons displayed impairments in dendritic outgrowth and branching (Fig. 6). These results are comparable with studies reported using neurons from Sema3A and CRMP1 knock-out mice where similar deficits in dendritic development were also observed (Schlomann et al., 2009; Makihara et al., 2016). Of note, dendritic cargoes, including the GluA2 subunit, were also identified on FEZ1 transport vesicles isolated from rat brains (Butkevich et al., 2016). Interestingly, activation of the Sema3A signaling pathway in axonal growth cones can stimulate the retrograde transport of PlxnA4, which mediates the dendritic localization of GluA2 subunits for dendritic development (Yamashita et al., 2014). A limitation in this study was the apparent stability of FEZ1 which prevented us from studying the impact of FEZ1 loss at earlier stages of development. Nevertheless, the observations here are likely to underestimate the actual importance of FEZ1 during initial neuronal development and we hypothesize that an earlier loss of FEZ1 is likely to produce even more acute developmental abnormalities.

Curiously, given that CRMP1 is an effector of Sema3A and the latter’s effect on suppressing axon development, our finding that axon development is impaired in FEZ1-deficient neurons is surprising if the adapter were to act solely downstream of the Sema3A pathway (Shelly et al., 2011). Additionally, the more severe dendritic branching defects observed in FEZ1-deficient neurons as compared with CRMP1-deficient neurons also suggested that other signaling pathways could be involved in regulating the function of the adapter during neurite extension (Fig. 6*B*,*D*). The colocalization and interaction of FEZ1 and VAMP2, a common SNARE effector in both Sema3A and Netrin-1 signaling pathways in developing neurites and growth cones, implicate the possible involvement of the latter in this regard (Bouchard et al., 2008; Zylbersztejn et al., 2012; Urbina et al., 2018). Indeed, our finding that a ternary complex of FEZ1, Stx1 and the Netrin-1 receptor DCC could be formed strongly supports this hypothesis. Taken together with FEZ1’s interaction with Nrp1 and PlxnA1, the results provide some insight as to why FEZ1 deficiency in developing neurons cause Netrin-1 and Sema3A insensitivity.

Netrin-1 is one of the most studied guidance cues; it plays roles not only in axonal guidance, but also axonal branching, dendritic development and synaptogenesis (Goldman et al., 2013; Winkle et al., 2014; Nagel et al., 2015). Exposure to Netrin-1 induces a VAMP2-dependent aggregation of DCC receptors to the surface membrane (Gopal et al., 2017), where it forms a complex with the SNARE proteins Stx1 and VAMP7 for exocytosis to achieve Netrin-1-induced attraction (Cotrufo et al., 2011). Although Stx1, VAMP2, and VAMP7 were all identified as cargoes present in FEZ1 and Kinesin-1-containing vesicles (Chua et al., 2012; Butkevich et al., 2016), DCC was surprisingly not included, suggesting that it may not be part of the transported cargoes. Rather, the interaction between DCC, Stx1 and FEZ1 might only occur at growth cones to mediate Netrin-1 signaling.

Importantly, when FEZ1-deficient and CRMP1-deficient neurons were treated with either Netrin-1 or Sema3A, only FEZ1-deficient neurons were unresponsive to both guidance cues with regards to dendrite development and unresponsive to Netrin-1 with regards to axon development. CRMP1-deficient neurons, on the other hand, responded only to Netrin-1 but not Sema3A as expected (Fig. 9). We did not observe changes in axonal branching when neurons in the control groups were exposed to either Netrin-1 or Sema3A, as opposed to previous reports (Dent et al., 2004; Winkle et al., 2014; Matsumoto and Nagashima, 2017). One possibility could be that the neurons used in previous studies were treated at an earlier time point (between days 2 and 5). In our case, treatment could only be applied after sufficient knock down of FEZ1 or CRMP1 is achieved (7 DIV), which may be too late for any effect on axons. Despite this, there was a significant increase in total axon length and longest neurite length for CRMP1-deficient neurons treated with Netrin-1. This is in agreement with other reports that Netrin-1 can increase axonal branching without affecting axon length (Dent et al., 2004; Winkle et al., 2014) and also increase in axon length (Barallobre et al., 2000; Bouchard et al., 2008; Matsumoto and Nagashima, 2017). Possibly, the stimulatory effects of Netrin-1 become more prominent in neurons lacking CRMP1 since their axons are less developed as compared with wild-type controls.

Given that FEZ1 can interact with components of Netrin-1 and Sema3A pathways and is required for their proper signaling (Figs. 8, 9), the concomitant defects in axon and dendrite development in FEZ1-deficient neurons is particularly interesting. In *Caenorhabditis elegans*, the nervous system is particularly compact, where most neurons are unbranched or have limited branching. An exception is the PVD somatosensory neuron which undergoes higher order dendritic branching (Sundararajan et al., 2019). Previously, it was found that Netrin mutants displayed a higher incidence of overlapping tertiary dendrites of the PVD neuron, suggesting that Netrin is required for proper self-avoidance of tertiary dendrites to maximize field coverage and prevent short circuits (Smith et al., 2012; Dong et al., 2015). More recently, it was found that Netrin also indirectly influences the extension and guidance of primary dendrites of the PVD neuron as a result of impaired axonal (ALA) patterning (Ramirez-Suarez et al., 2019). In mice, Netrin-1 influences axonal branching through TRIM9, which regulates a VAMP2/SNAP25 interaction for membrane expansion (Winkle et al., 2014). While it is still unknown whether the dendritic phenotypes arising from axonal defects observed in *C. elegans* are conserved in higher order organisms, these studies raise an interesting possibility in that in neurons lacking FEZ1, dendritic defects may be exacerbated by aberrant axon development resulting from the disruption to Sema3A and Netrin-1 signaling. It would also be interesting to study whether FEZ1 associates with TRIM-9, which as aforementioned, regulates Netrin-1-induced axonal and dendritic branching (Winkle et al., 2014, 2016).

Netrin-1 and Sema3A and 3F have also been shown to work together during the formation of septo-hippocampal connections. Netrin-1 produced by the fimbria promote the growth of axons of the medial septum-diagonal band complex into the hippocampus. At the same time, surrounding regions express Sema3A and 3F, repelling axons non-target regions (Pascual et al., 2004). While we have shown that FEZ1 is required for Netrin-1-induced and Sema3A-induced axonal and dendritic development, our experimental set-up is insufficient to investigate exactly how FEZ1 is required for Netrin-1 and Sema3A guidance *in vivo*. These will be important areas for future studies.

## Synthesis

Reviewing Editor: Jeffery Twiss, University of South Carolina Decisions are customarily a result of the Reviewing Editor and the peer reviewers coming together and discussing their recommendations until a consensus is reached. When revisions are invited, a fact-based synthesis statement explaining their decision and outlining what is needed to prepare a revision will be listed below.

I apologize for the delays in getting an assessment back to you. Unfortunately, one of the previous reviewers was unable to assist this round, so the revision was reviewed by 1 previous reviewer and me. Our assessment derives both from our individual critiques and online discussion (you will see below there is some equivocation that came from the online discussion). The study is well written, and your group has gone a long way to address the concerns of the reviewers. We both felt that the manuscript is much improved, and the additional data support your conclusions. In particular, using both a CRISPR and a shRNA lentiviral approach show that like CRMP1 knockdown, FEZ1 knockdown decreases axonal length and branching in cultured hippocampal neurons. The FEZ1 knockdown also reduces dendritic branching to a larger extent than CRMP1 knockdown. FEZ1 co-localization with the synaptic vesicular SNARE VAMP2 in growth cones, and that FEZ1 forming a complex with the Netrin receptor DCC and Syntaxin-1 (Stx1) point to mechanisms of action. Further, the FEZ1-deficient neurons did not exhibit increased dendritic development in response to Netrin-1 or Sema3A, while CRMP1-knockdown only prevented the effects of Sema3A. Together, these results advance the field of developmental neurobiology, as they implicate FEZ1 as a hub regulating two guidance signaling pathways important for axonal and dendritic development. The demonstration that FEZ1 knockdown blocks the effect of Sema3a and Netrin on dendrites is a major point of the study, and the use of an shRNA lentiviral approach to confirm the results observed after CRISPR-mediated knockdown of FEZ1 is appropriate. However, we both felt that the impact of the manuscript would be strengthened by addressing a few points:

1) The authors show in their response to the reviewers that FEZ1 interacts with VAMP2 and the Nrp1/PLxnA1 complex (was mCherry immunoprecipitated in that experiment?). These results that were included in your rebuttal should be added to the manuscript (possibly in figure 8), as they provide a mechanism as for why FEZ1 depletion blocks the effects of Sema3A on dendritic development. These results also confirm the hypothesis that FEZ1 is indeed a common integrator of Sema3A and Netrin-1 pathways. Of course, we presume the co-IP data shown are replicated and not pilot experiments. Please note that neither the reviewer nor me were clear on how the experiment in the second blot was performed, specifically what was precipitated. So, if we are off the mark on interpreting that it complements the data showing FEZ1 and DCC interaction, please let me know that in revision.

2) For Figure 9, the authors mention in their text that they intriguingly did not observe any effect of Sema3A or Netrin1 on control axons. However, that lack of effect is not that surprising considering that treatments were applied at 6 days post infection, well after axons have developed in culture. In contrast, Netrin-1 had effects on neurons lacking CRMP1 because their axons do not develop as fast as controls. The author should raise that point in their text, either in the results or discussion (or both).

3) Also, for Figure 9, since Netrin-1 and Sema3A have no effect on axons in control conditions, it is only by comparing FEZ-depleted axons to CRMP1-depleted axons that the authors can conclude about a role of FEZ1 downstream of Netrin-1. That point should be better explained in the text (lines 580-586). In addition, the author should rephrase the sentence in their discussion stating that “developing axons and dendrites of FEZ-1-deficient neurons failed to respond to Sema3A and Netrin-1 stimulation” (lines 601-603), since control axons failed to respond to Sema3A and Netrin-1 as well (a role downstream of Sema3A in axonal development has not been demonstrated).

Minor points:

- line 307: a space is missing between “HEK293” and “cells”.

- line 414: a word is missing after “successfully”.

- line 554: “proliferation” is not confusing as dendrites do not proliferate. So please be more specific in your wording.

- line 555: please change “looked at” to “tested”.

## References

[B1] Barallobre MJ, Del Rio JA, Alcantara S, Borrell V, Aguado F, Ruiz M, Carmona MA, Martin M, Fabre M, Yuste R, Tessier-Lavigne M, Soriano E (2000) Aberrant development of hippocampal circuits and altered neural activity in netrin 1-deficient mice. Development 127:4797–4810. 11044395 10.1242/dev.127.22.4797

[B2] Barrecheguren PJ, Ros O, Cotrufo T, Kunz B, Soriano E, Ulloa F, Stoeckli ET, Araújo SJ (2017) SNARE proteins play a role in motor axon guidance in vertebrates and invertebrates. Dev Neurobiol 77:963–974. 10.1002/dneu.22481 28033683

[B3] Barszczewski M, Chua JJ, Stein A, Winter U, Heintzmann R, Zilly FE, Fasshauer D, Lang T, Jahn R (2008) A novel site of action for alpha-SNAP in the SNARE conformational cycle controlling membrane fusion. Mol Biol Cell 19:776–784. 10.1091/mbc.e07-05-0498 18094056 PMC2262999

[B4] Baumert M, Maycox PR, Navone F, De Camilli P, Jahn R (1989) Synaptobrevin: an integral membrane protein of 18,000 daltons present in small synaptic vesicles of rat brain. EMBO J 8:379–384. 2498078 10.1002/j.1460-2075.1989.tb03388.x PMC400817

[B5] Bilimoria PM, Bonni A (2013) Molecular control of axon branching. The Neuroscientist: a review journal bringing neurobiology. Neuroscientist 19:16–24. 10.1177/1073858411426201 22179123 PMC3490022

[B6] Biswas S, Kalil K (2018) The microtubule-associated protein tau mediates the organization of microtubules and their dynamic exploration of actin-rich lamellipodia and filopodia of cortical growth cones. J Neurosci 38:291–307. 10.1523/JNEUROSCI.2281-17.2017 29167405 PMC5761611

[B7] Bouchard JF, Horn KE, Stroh T, Kennedy TE (2008) Depolarization recruits DCC to the plasma membrane of embryonic cortical neurons and enhances axon extension in response to netrin-1. J Neurochem 107:398–417. 10.1111/j.1471-4159.2008.05609.x 18691385

[B8] Butkevich E, Härtig W, Nikolov M, Erck C, Grosche J, Urlaub H, Schmidt CF, Klopfenstein DR, Chua JJ (2016) Phosphorylation of FEZ1 by microtubule affinity regulating kinases regulates its function in presynaptic protein trafficking. Sci Rep 6:26965. 10.1038/srep26965 27247180 PMC4887895

[B9] Cai Q, Pan PY, Sheng ZH (2007) Syntabulin-kinesin-1 family member 5B-mediated axonal transport contributes to activity-dependent presynaptic assembly. J Neurosci 27:7284–7296. 10.1523/JNEUROSCI.0731-07.2007 17611281 PMC6794594

[B10] Chua JJ, Butkevich E, Worseck JM, Kittelmann M, Grønborg M, Behrmann E, Stelzl U, Pavlos NJ, Lalowski MM, Eimer S, Wanker EE, Klopfenstein DR, Jahn R (2012) Phosphorylation-regulated axonal dependent transport of syntaxin 1 is mediated by a Kinesin-1 adapter. Proc Natl Acad Sci USA 109:5862–5867. 10.1073/pnas.1113819109 22451907 PMC3326461

[B11] Coco S, Raposo G, Martinez S, Fontaine JJ, Takamori S, Zahraoui A, Jahn R, Matteoli M, Louvard D, Galli T (1999) Subcellular localization of tetanus neurotoxin-insensitive vesicle-associated membrane protein (VAMP)/VAMP7 in neuronal cells: evidence for a novel membrane compartment. J Neurosci 19:9803–9812. 10.1523/JNEUROSCI.19-22-09803.1999 10559389 PMC6782963

[B12] Concordet J-P, Haeussler M (2018) CRISPOR: intuitive guide selection for CRISPR/Cas9 genome editing experiments and screens. Nucleic Acids Res 46:W242–W245. 10.1093/nar/gky354 29762716 PMC6030908

[B13] Cotrufo T, Pérez-Brangulí F, Muhaisen A, Ros O, Andrés R, Baeriswyl T, Fuschini G, Tarrago T, Pascual M, Ureña J, Blasi J, Giralt E, Stoeckli ET, Soriano E (2011) A signaling mechanism coupling netrin-1/deleted in colorectal cancer chemoattraction to SNARE-mediated exocytosis in axonal growth cones. J Neurosci 31:14463–14480. 10.1523/JNEUROSCI.3018-11.2011 21994363 PMC6703395

[B14] Dent EW, Barnes AM, Tang F, Kalil K (2004) Netrin-1 and semaphorin 3A promote or inhibit cortical axon branching, respectively, by reorganization of the cytoskeleton. J Neurosci 24:3002–3012. 10.1523/JNEUROSCI.4963-03.2004 15044539 PMC6729836

[B15] Deo RC, Schmidt EF, Elhabazi A, Togashi H, Burley SK, Strittmatter SM (2004) Structural bases for CRMP function in plexin-dependent semaphorin3A signaling. EMBO J 23:9–22. 10.1038/sj.emboj.7600021 14685275 PMC1271659

[B16] Dong X, Shen K, Bülow HE (2015) Intrinsic and extrinsic mechanisms of dendritic morphogenesis. Annu Rev Physiol 77:271–300. 10.1146/annurev-physiol-021014-071746 25386991

[B17] Fournier AE, Nakamura F, Kawamoto S, Goshima Y, Kalb RG, Strittmatter SM (2000) Semaphorin3A enhances endocytosis at sites of receptor-F-actin colocalization during growth cone collapse. J Cell Biol 149:411–422. 10.1083/jcb.149.2.411 10769032 PMC2175148

[B18] Fujita T, Maturana AD, Ikuta J, Hamada J, Walchli S, Suzuki T, Sawa H, Wooten MW, Okajima T, Tatematsu K, Tanizawa K, Kuroda S (2007) Axonal guidance protein FEZ1 associates with tubulin and kinesin motor protein to transport mitochondria in neurites of NGF-stimulated PC12 cells. Biochem Biophys Res Commun 361:605–610. 10.1016/j.bbrc.2007.07.050 17669366

[B19] Gindhart JG, Chen J, Faulkner M, Gandhi R, Doerner K, Wisniewsk T, Nandlestadt A (2003) The kinesin-associated protein UNC-76 is required for axonal transport in the *Drosophila* nervous system. Mol Biol Cell 14:3356–3365. 10.1091/mbc.e02-12-0800 12925768 PMC181572

[B20] Goldman JS, Ashour MA, Magdesian MH, Tritsch NX, Harris SN, Christofi N, Chemali R, Stern YE, Thompson-Steckel G, Gris P, Glasgow SD, Grutter P, Bouchard JF, Ruthazer ES, Stellwagen D, Kennedy TE (2013) Netrin-1 promotes excitatory synaptogenesis between cortical neurons by initiating synapse assembly. J Neurosci 33:17278–17289. 10.1523/JNEUROSCI.1085-13.2013 24174661 PMC6618363

[B21] Gopal AA, Ricoult SG, Harris SN, Juncker D, Kennedy TE, Wiseman PW (2017) Spatially selective dissection of signal transduction in neurons grown on Netrin-1 printed nanoarrays via segmented fluorescence fluctuation analysis. ACS nano 11:8131–8143. 10.1021/acsnano.7b03004 28679208

[B22] Haeussler M, Schönig K, Eckert H, Eschstruth A, Mianné J, Renaud JB, Schneider-Maunoury S, Shkumatava A, Teboul L, Kent J, Joly JS, Concordet JP (2016) Evaluation of off-target and on-target scoring algorithms and integration into the guide RNA selection tool CRISPOR. Genome Biol 17:148. 10.1186/s13059-016-1012-2 27380939 PMC4934014

[B23] Higurashi M, Iketani M, Takei K, Yamashita N, Aoki R, Kawahara N, Goshima Y (2012) Localized role of CRMP1 and CRMP2 in neurite outgrowth and growth cone steering. Dev Neurobiol 72:1528–1540. 10.1002/dneu.22017 22378692

[B24] Hirokawa N, Takemura R (2005) Molecular motors and mechanisms of directional transport in neurons. Nat Rev Neurosci 6:201–214. 10.1038/nrn1624 15711600

[B25] Huang H, Shao Q, Qu C, Yang T, Dwyer T, Liu G (2015) Coordinated interaction of Down syndrome cell adhesion molecule and deleted in colorectal cancer with dynamic TUBB3 mediates Netrin-1-induced axon branching. Neuroscience 293:109–122. 10.1016/j.neuroscience.2015.02.042 25754961 PMC4386621

[B26] Ikuta J, Maturana A, Fujita T, Okajima T, Tatematsu K, Tanizawa K, Kuroda S (2007) Fasciculation and elongation protein zeta-1 (FEZ1) participates in the polarization of hippocampal neuron by controlling the mitochondrial motility. Biochem Biophys Res Commun 353:127–132. 10.1016/j.bbrc.2006.11.142 17173861

[B27] Kang E, Burdick KE, Kim JY, Duan X, Guo JU, Sailor KA, Jung DE, Ganesan S, Choi S, Pradhan D, Lu B, Avramopoulos D, Christian K, Malhotra AK, Song H, Ming GL (2011) Interaction between FEZ1 and DISC1 in regulation of neuronal development and risk for schizophrenia. Neuron 72:559–571. 10.1016/j.neuron.2011.09.032 22099459 PMC3222865

[B28] Keino-Masu K, Masu M, Hinck L, Leonardo ED, Chan SS, Culotti JG, Tessier-Lavigne M (1996) Deleted in colorectal cancer (DCC) encodes a netrin receptor. Cell 87:175–185. 10.1016/s0092-8674(00)81336-7 8861902

[B29] Kennedy TE, Serafini T, de la Torre JR, Tessier-Lavigne M (1994) Netrins are diffusible chemotropic factors for commissural axons in the embryonic spinal cord. Cell 78:425–435. 10.1016/0092-8674(94)90421-9 8062385

[B30] Kim S, Chiba A (2004) Dendritic guidance. Trends Neurosci 27:194–202. 10.1016/j.tins.2004.02.011 15046878

[B31] Kolodkin AL, Tessier-Lavigne M (2011) Mechanisms and molecules of neuronal wiring: a primer. Cold Spring Harbor Perspect Biol 3:a001727. 10.1101/cshperspect.a001727 PMC3098670 21123392

[B32] Kurochkina N (2010) Helix-helix interactions and their impact on protein motifs and assemblies. J Theor Biol 264:585–592. 10.1016/j.jtbi.2010.02.026 20202472

[B33] Kuroda S, Nakagawa N, Tokunaga C, Tatematsu K, Tanizawa K (1999) Mammalian homologue of the *Caenorhabditis elegans* UNC-76 protein involved in axonal outgrowth is a protein kinase C zeta-interacting protein. J Cell Biol 144:403–411. 10.1083/jcb.144.3.403 9971736 PMC2132904

[B34] Lanoue V, Cooper HM (2019) Branching mechanisms shaping dendrite architecture. Dev Biol 451:16–24. 10.1016/j.ydbio.2018.12.005 30550882

[B35] Luo Y, Raible D, Raper JA (1993) Collapsin: a protein in brain that induces the collapse and paralysis of neuronal growth cones. Cell 75:217–227. 10.1016/0092-8674(93)80064-l 8402908

[B36] Ly A, Nikolaev A, Suresh G, Zheng Y, Tessier-Lavigne M, Stein E (2008) DSCAM is a netrin receptor that collaborates with DCC in mediating turning responses to netrin-1. Cell 133:1241–1254. 10.1016/j.cell.2008.05.030 18585357 PMC2491333

[B37] Makihara H, Nakai S, Ohkubo W, Yamashita N, Nakamura F, Kiyonari H, Shioi G, Jitsuki-Takahashi A, Nakamura H, Tanaka F, Akase T, Kolattukudy P, Goshima Y (2016) CRMP1 and CRMP2 have synergistic but distinct roles in dendritic development. Genes Cells 21:994–1005. 10.1111/gtc.12399 27480924

[B38] Mandell JW, Banker GA (1996) A spatial gradient of tau protein phosphorylation in nascent axons. J Neurosci 16:5727–5740. 10.1523/JNEUROSCI.16-18-05727.1996 8795628 PMC6578967

[B39] Matsumoto H, Nagashima M (2017) Shift in the function of netrin-1 from axon outgrowth to axon branching in developing cerebral cortical neurons. BMC Neurosci 18:74. 10.1186/s12868-017-0392-x 29041904 PMC5645936

[B40] Messersmith EK, Leonardo ED, Shatz CJ, Tessier-Lavigne M, Goodman CS, Kolodkin AL (1995) Semaphorin III can function as a selective chemorepellent to pattern sensory projections in the spinal cord. Neuron 14:949–959. 10.1016/0896-6273(95)90333-x 7748562

[B41] Nagel AN, Marshak S, Manitt C, Santos RA, Piercy MA, Mortero SD, Shirkey-Son NJ, Cohen-Cory S (2015) Netrin-1 directs dendritic growth and connectivity of vertebrate central neurons in vivo. Neural Dev 10:14. 10.1186/s13064-015-0041-y 26058786 PMC4481067

[B42] Niquille M, Garel S, Mann F, Hornung JP, Otsmane B, Chevalley S, Parras C, Guillemot F, Gaspar P, Yanagawa Y, Lebrand C (2009) Transient neuronal populations are required to guide callosal axons: a role for semaphorin 3C. PLoS Biol 7:e1000230. 10.1371/journal.pbio.1000230 19859539 PMC2762166

[B43] Pascual M, Pozas E, Barallobre MJ, Tessier-Lavigne M, Soriano E (2004) Coordinated functions of Netrin-1 and Class 3 secreted Semaphorins in the guidance of reciprocal septohippocampal connections. Mol Cell Neurosci 26:24–33. 10.1016/j.mcn.2003.12.008 15121176

[B44] Pfenninger KH (2009) Plasma membrane expansion: a neuron’s Herculean task. Nat Rev Neurosci 10:251–261. 10.1038/nrn2593 19259102

[B45] Ramirez-Suarez NJ, Belalcazar HM, Salazar CJ, Beyaz B, Raja B, Nguyen KCQ, Celestrin K, Fredens J, Færgeman NJ, Hall DH, Bülow HE (2019) Axon-dependent patterning and maintenance of somatosensory dendritic arbors. Dev Cell 48:229–244.e4. 10.1016/j.devcel.2018.12.015 30661986 PMC6442679

[B46] Rohm B, Ottemeyer A, Lohrum M, Püschel AW (2000) Plexin/neuropilin complexes mediate repulsion by the axonal guidance signal semaphorin 3A. Mech Dev 93:95–104. 10.1016/s0925-4773(00)00269-0 10781943

[B47] Sakae N, Yamasaki N, Kitaichi K, Fukuda T, Yamada M, Yoshikawa H, Hiranita T, Tatsumi Y, Kira J, Yamamoto T, Miyakawa T, Nakayama KI (2008) Mice lacking the schizophrenia-associated protein FEZ1 manifest hyperactivity and enhanced responsiveness to psychostimulants. Hum Mol Genet 17:3191–3203. 10.1093/hmg/ddn215 18647754

[B48] Sanjana NE, Shalem O, Zhang F (2014) Improved vectors and genome-wide libraries for CRISPR screening. Nat Methods 11:783–784. 10.1038/nmeth.3047 25075903 PMC4486245

[B49] Schlomann U, Schwamborn JC, Müller M, Fässler R, Püschel AW (2009) The stimulation of dendrite growth by Sema3A requires integrin engagement and focal adhesion kinase. J Cell Sci 122:2034–2042. 10.1242/jcs.038232 19454481

[B50] Serafini T, Kennedy TE, Galko MJ, Mirzayan C, Jessell TM, Tessier-Lavigne M (1994) The netrins define a family of axon outgrowth-promoting proteins homologous to *C. elegans* UNC-6. Cell 78:409–424. 10.1016/0092-8674(94)90420-0 8062384

[B51] Shalem O, Sanjana NE, Hartenian E, Shi X, Scott DA, Mikkelson T, Heckl D, Ebert BL, Root DE, Doench JG, Zhang F (2014) Genome-scale CRISPR-Cas9 knockout screening in human cells. Science 343:84–87. 10.1126/science.1247005 24336571 PMC4089965

[B52] Shelly M, Cancedda L, Lim BK, Popescu AT, Cheng PL, Gao H, Poo MM (2011) Semaphorin3A regulates neuronal polarization by suppressing axon formation and promoting dendrite growth. Neuron 71:433–446. 10.1016/j.neuron.2011.06.041 21835341 PMC3164872

[B53] Smith CJ, Watson JD, VanHoven MK, Colón-Ramos DA, Miller DM 3rd (2012) Netrin (UNC-6) mediates dendritic self-avoidance. Nat Neurosci 15:731–737. 10.1038/nn.3065 22426253 PMC3337961

[B54] Stauffer W, Sheng H, Lim HN (2018) EzColocalization: an ImageJ plugin for visualizing and measuring colocalization in cells and organisms. Sci Rep 8:15764. 10.1038/s41598-018-33592-8 30361629 PMC6202351

[B55] Stoeckli ET (2018) Understanding axon guidance: are we nearly there yet? Development 145:dev151415. 10.1242/dev.151415 29759980

[B56] Su KY, Chien WL, Fu WM, Yu IS, Huang HP, Huang PH, Lin SR, Shih JY, Lin YL, Hsueh YP, Yang PC, Lin SW (2007) Mice deficient in collapsin response mediator protein-1 exhibit impaired long-term potentiation and impaired spatial learning and memory. J Neurosci 27:2513–2524. 10.1523/JNEUROSCI.4497-06.2007 17344389 PMC6672508

[B57] Su Q, Cai Q, Gerwin C, Smith CL, Sheng ZH (2004) Syntabulin is a microtubule-associated protein implicated in syntaxin transport in neurons. Nat Cell Biol 6:941–953. 10.1038/ncb1169 15459722

[B58] Sun F, Zhu C, Dixit R, Cavalli V (2011) Sunday Driver/JIP3 binds kinesin heavy chain directly and enhances its motility. EMBO J 30:3416–3429. 10.1038/emboj.2011.229 21750526 PMC3160654

[B59] Sundararajan L, Stern J, Miller DM 3rd (2019) Mechanisms that regulate morphogenesis of a highly branched neuron in *C. elegans*. Dev Biol 451:53–67. 10.1016/j.ydbio.2019.04.002 31004567 PMC7755292

[B60] Takahashi T, Fournier A, Nakamura F, Wang LH, Murakami Y, Kalb RG, Fujisawa H, Strittmatter SM (1999) Plexin-neuropilin-1 complexes form functional semaphorin-3A receptors. Cell 99:59–69. 10.1016/s0092-8674(00)80062-8 10520994

[B61] Toda H, Mochizuki H, Flores R 3rd, Josowitz R, Krasieva TB, Lamorte VJ, Suzuki E, Gindhart JG, Furukubo-Tokunaga K, Tomoda T (2008) UNC-51/ATG1 kinase regulates axonal transport by mediating motor-cargo assembly. Genes Dev 22:3292–3307. 10.1101/gad.1734608 19056884 PMC2600757

[B62] Ulloa F, Cotrufo T, Ricolo D, Soriano E, Araújo SJ (2018) SNARE complex in axonal guidance and neuroregeneration. Neural Regen Res 13:386–392. 10.4103/1673-5374.228710 29623913 PMC5900491

[B63] Urbina FL, Gomez SM, Gupton SL (2018) Spatiotemporal organization of exocytosis emerges during neuronal shape change. J Cell Biol 217:1113–1128. 10.1083/jcb.201709064 29351997 PMC5839795

[B64] Watanabe Y, Khodosevich K, Monyer H (2014) Dendrite development regulated by the schizophrenia-associated gene FEZ1 involves the ubiquitin proteasome system. Cell Rep 7:552–564. 10.1016/j.celrep.2014.03.022 24726361

[B65] Watt D, Dixit R, Cavalli V (2015) JIP3 activates kinesin-1 motility to promote axon elongation. J Biol Chem 290:15512–15525. 10.1074/jbc.M115.651885 25944905 PMC4505465

[B66] Winkle CC, McClain LM, Valtschanoff JG, Park CS, Maglione C, Gupton SL (2014) A novel Netrin-1-sensitive mechanism promotes local SNARE-mediated exocytosis during axon branching. J Cell Biol 205:217–232. 10.1083/jcb.201311003 24778312 PMC4003241

[B67] Winkle CC, Olsen RH, Kim H, Moy SS, Song J, Gupton SL (2016) Trim9 deletion alters the morphogenesis of developing and adult-born hippocampal neurons and impairs spatial learning and memory. J Neurosci 36:4940–4958. 10.1523/JNEUROSCI.3876-15.2016 27147649 PMC4854964

[B68] Wojnacki J, Galli T (2016) Membrane traffic during axon development. Dev Neurobiol 76:1185–1200. 10.1002/dneu.22390 26945675

[B69] Yagensky O, Kohansal-Nodehi M, Gunaseelan S, Rabe T, Zafar S, Zerr I, Härtig W, Urlaub H, Chua JJ (2019) Increased expression of heme-binding protein 1 early in Alzheimer’s disease is linked to neurotoxicity. Elife 8:e47498. 10.7554/eLife.47498 31453805 PMC6739868

[B70] Yamashita N, Goshima Y (2012) Collapsin response mediator proteins regulate neuronal development and plasticity by switching their phosphorylation status. Mol Neurobiol 45:234–246. 10.1007/s12035-012-8242-4 22351471

[B71] Yamashita N, Morita A, Uchida Y, Nakamura F, Usui H, Ohshima T, Taniguchi M, Honnorat J, Thomasset N, Takei K, Takahashi T, Kolattukudy P, Goshima Y (2007) Regulation of spine development by semaphorin3A through cyclin-dependent kinase 5 phosphorylation of collapsin response mediator protein 1. J Neurosci 27:12546–12554. 10.1523/JNEUROSCI.3463-07.2007 18003833 PMC6673320

[B72] Yamashita N, Usui H, Nakamura F, Chen S, Sasaki Y, Hida T, Suto F, Taniguchi M, Takei K, Goshima Y (2014) Plexin-A4-dependent retrograde semaphorin 3A signalling regulates the dendritic localization of GluA2-containing AMPA receptors. Nat Commun 5:3424. 10.1038/ncomms4424 24599038

[B73] Zylbersztejn K, Petkovic M, Burgo A, Deck M, Garel S, Marcos S, Bloch-Gallego E, Nothias F, Serini G, Bagnard D, Binz T, Galli T (2012) The vesicular SNARE Synaptobrevin is required for Semaphorin 3A axonal repulsion. J Cell Biol 196:37–46. 10.1083/jcb.201106113 22213797 PMC3255983

