# Supplementary figures and images for: FEZ1 Forms Complexes with CRMP1 and DCC to Regulate Axon and Dendrite Development
Source: eNeuro. 2021 Apr 15;8(2):ENEURO.0193-20.2021. doi: 10.1523/ENEURO.0193-20.2021 (PMC8174033; doi:10.1523/ENEURO.0193-20.2021)

**Extended Data Figure 1-1
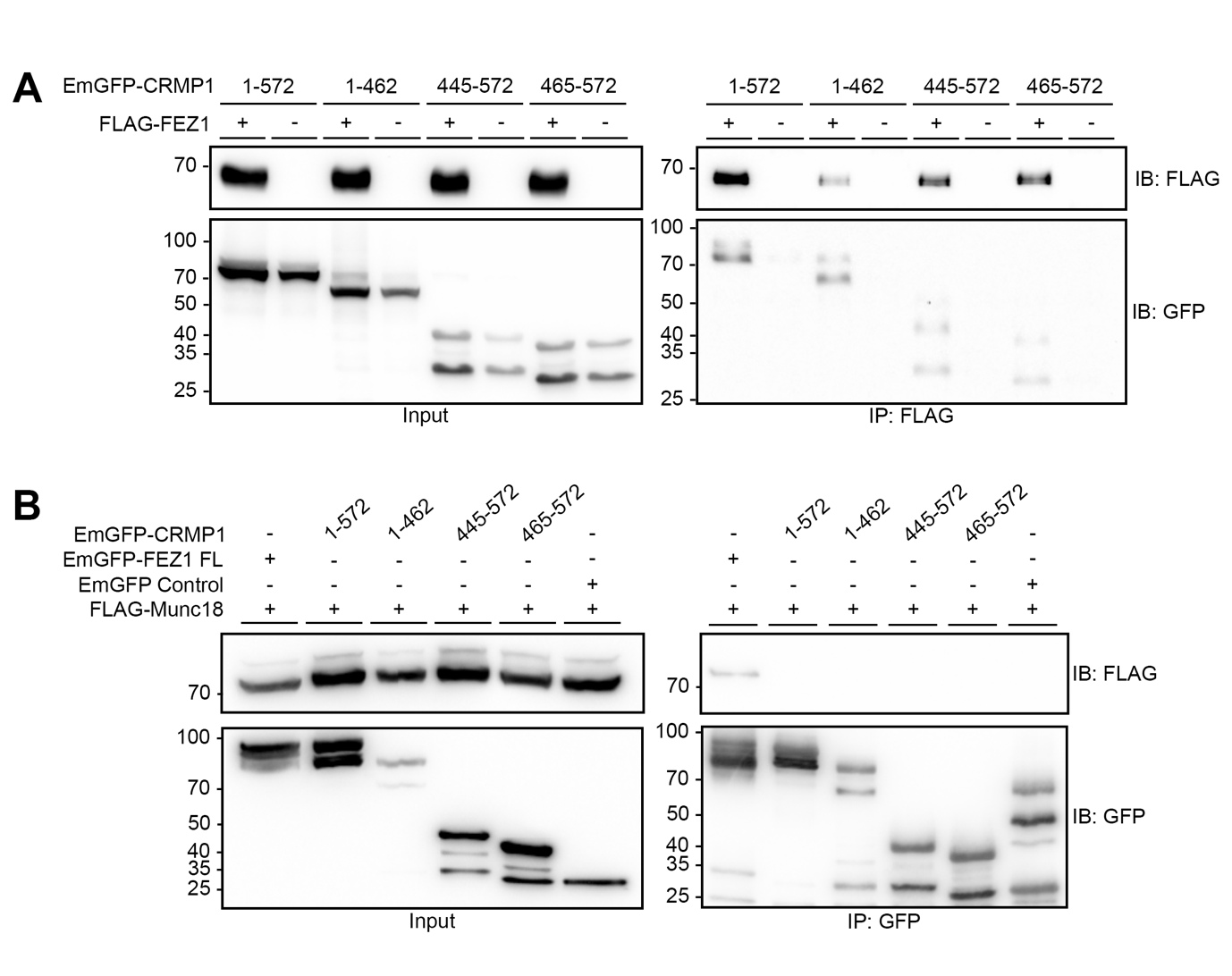
**

Supplement: Extended Data Figure 1-1 — Reciprocal coimmunoprecipitations for FEZ1 and CRMP1 interaction. A, Reciprocal co-immunoprecipitation of EmGFP-CRMP1 truncated constructs. Cell lysates from HEK293 cells transiently expressing FLAG-FEZ1 and various EmGFP-tagged CRMP1 peptides were immunoprecipitated using α-FLAG. Both the upper and lower bands of each EmGFP-CRMP1 construct were co-immunoprecipitated and were absent in their corresponding control coIPs. B, HEK293 cell lysates containing FLAG-Munc18 co-expressing EmGFP-FEZ1 FL or various EmGFP-tagged CRMP1 peptides were immunoprecipitated using GFP-TRAP beads. Only the positive control, EmGFP-FEZ1 FL, was observed to interact with FLAG-Munc18. Download Figure 1-1, DOCX file. [file enu-eN-NWR-0193-20-s01.docx]

**Extended Data Figure 3-1
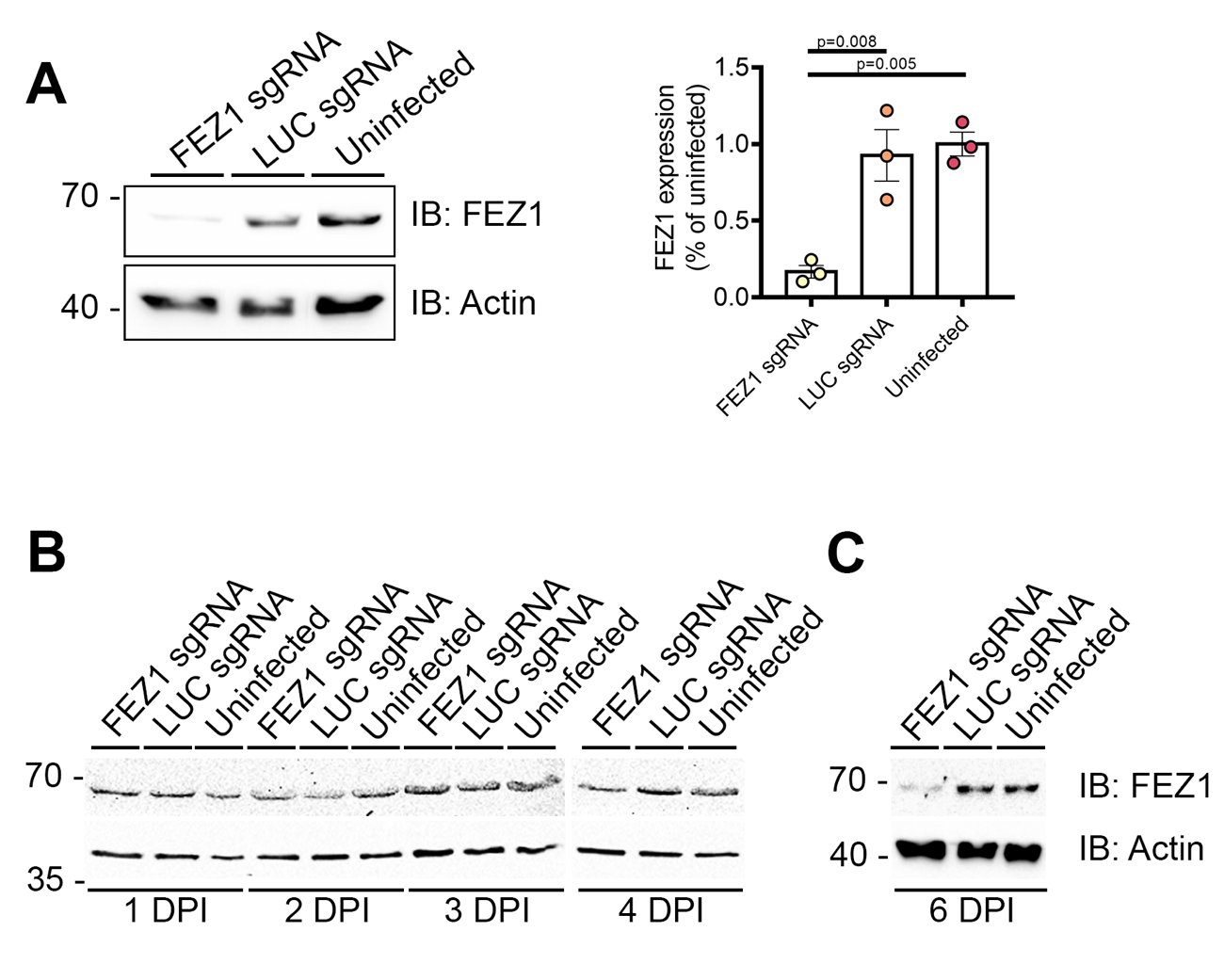
re 3-1**

Supplement: Extended Data Figure 3-1 — Knock down of FEZ1 in rat hippocampal neurons. The Lentiviral CRISPR/Cas9 system was used to knock-down FEZ1 in primary rat hippocampal neurons at DIV1. A, Representative Western blotting showing FEZ1 knock-down in rat hippocampal neurons 6 DPI. FEZ1 levels was reduced by ∼70%. Statistical significance was determined using one-way ANOVA. Data were obtained from three independent experiments. Values displayed represent mean ± SEM. B, Neurons at 1–4 DPI were lysed and probed using an α-FEZ1 antibody. No changes in FEZ1 levels compared to control neurons were observed from 1 to 4 DPI. C, However, at 6 DPI, a clear decrease in FEZ1 levels were observed. Download Figure 3-1, DOCX file. [file enu-eN-NWR-0193-20-s02.docx]

**Extended Data Figure 4-1**

**
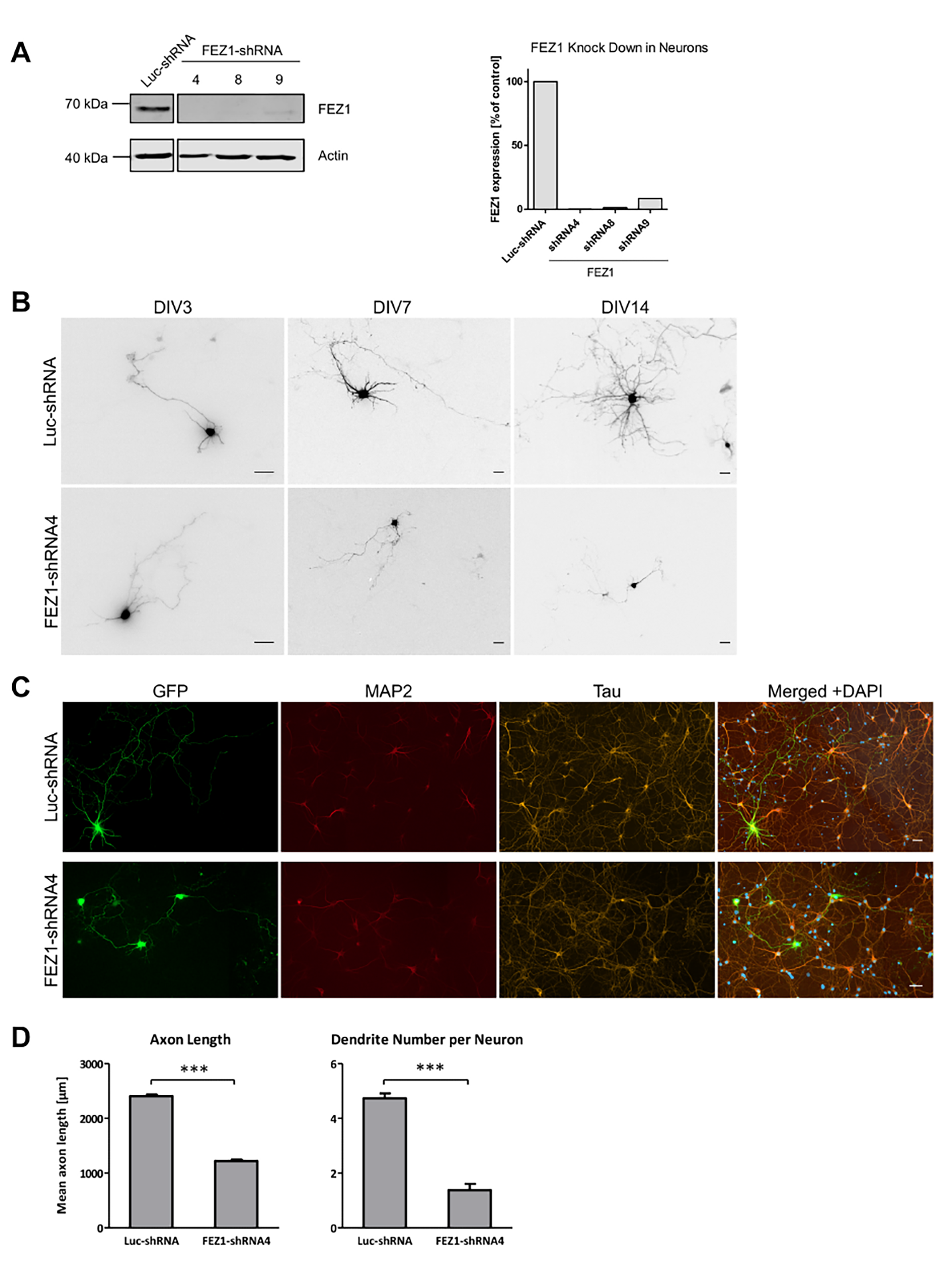
**

Supplement: Extended Data Figure 4-1 — shRNA-mediated FEZ1 knock-down in rat hippocampal neurons. A, Representative Western blotting showing FEZ1 knock-down in rat hippocampal neurons. Quantification is shown on the right. B, C, FEZ1 knock-down neurons show axo-dendrite developmental abnormalities. D, Quantification of total axon length and dendrite branches. FEZ1 knock-down neurons show significantly shorter axons and dendrite branching as compared to control neurons. Download Figure 4-1, DOCX file. [file enu-eN-NWR-0193-20-s03.docx]

**Extended Data Figure 5-1**

**
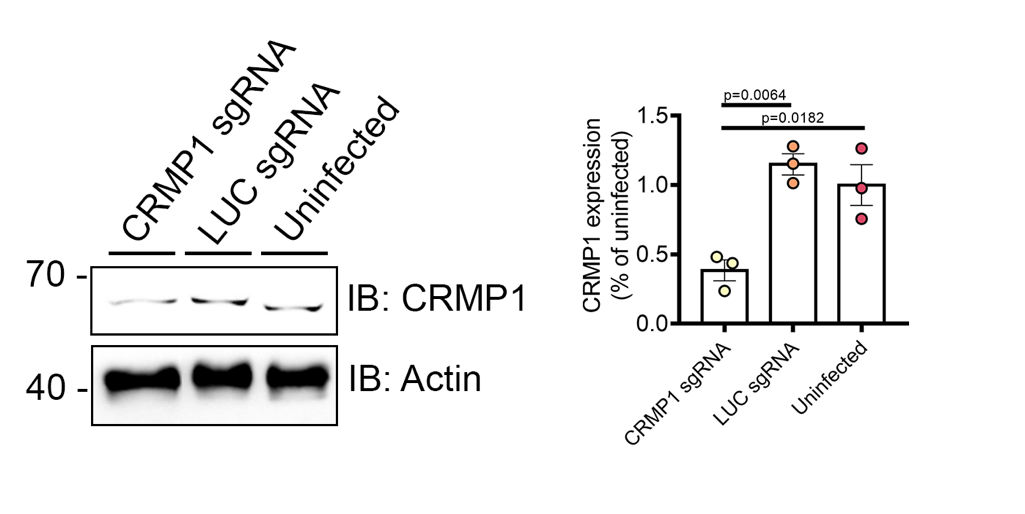
**

Supplement: Extended Data Figure 5-1 — Knock down of CRMP1 in rat hippocampal neurons. Representative Western blotting showing knock down of CRMP1 in rat hippocampal neurons 6 DPI. Approximately 60% of CRMP1 was eliminated. Statistical significance was determined using one-way ANOVA. Data were obtained from three independent experiments. Values displayed represent mean ± SEM. Download Figure 5-1, DOCX file. [file enu-eN-NWR-0193-20-s04.docx]

**Extended Data Figure 7-1**

**
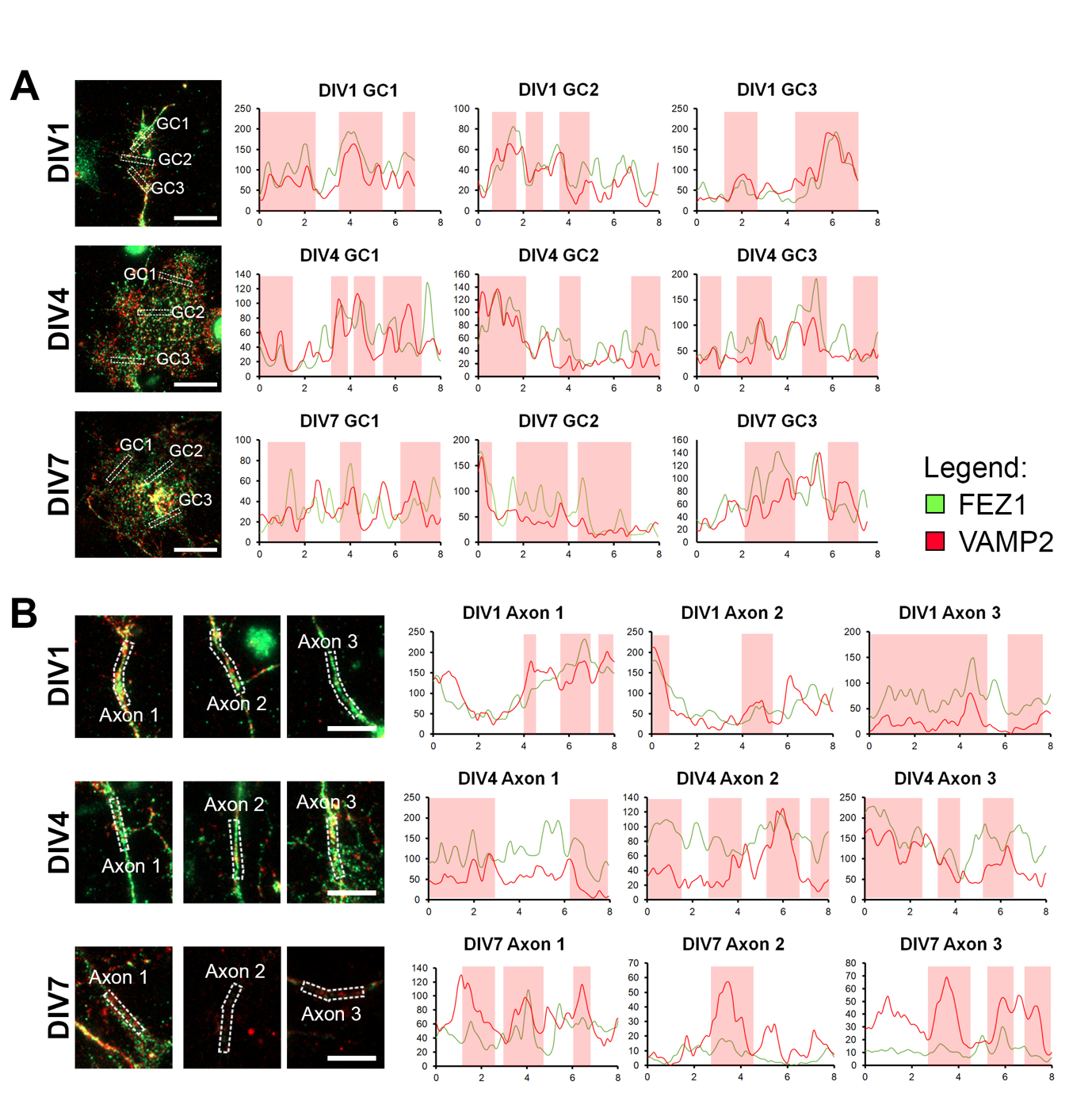
**

Supplement: Extended Data Figure 7-1 — Additional line scan analyses for FEZ1 and VAMP2 in growth cones and axons of developing neurons. A, B, Line scan analyses for growth cones and axons of neurons at 1, 4, and 7 DIV, respectively. Merged images are shown. x- and y-axes, distance (μm) and grey values, respectively; axon 1, distal; axon 2, intermediate; axon 3, proximal (relative to cell body). Vertical columns in red represent regions of colocalization. Scale bars: 10 μm (A) and 5 μm (B). Download Figure 7-1, DOCX file. [file enu-eN-NWR-0193-20-s05.docx]
